# Supplementary material for: Croargoids A–G, Eudesmane Sesquiterpenes from the Bark of Croton argyratus
Source: Molecules. 2022 Sep 27;27(19):6397. doi: 10.3390/molecules27196397 (PMC9571801; doi:10.3390/molecules27196397)
Supplement: Supplementary file 1 [file molecules-27-06397-s001.zip › molecules-1906435-supplementary.pdf]

molecules

Supplementary Materials

Croargoids A–G, Eudesmane Sesquiterpenes from the Bark of *Croton argyratus*

Min Wu [1,2], Kai-Long Ji [1], Peng Sun [1,2], Jian-Mei Lu [1,2], Jia-Rui Yue [1,3], Dong-Hua Cao [4], Chun-Fen Xiao [1] and You-Kai Xu [1,*]

[1] Key Laboratory of Tropical Plant Resources and Sustainable Use, Xishuangbanna Tropical Botanical Garden, Chinese Academy of Sciences, Yunnan 666303, China
[2] University of Chinese Academy of Sciences, Beijing 100049, China
[3] School of Pharmaceutical Science and Yunnan Key Laboratory of Pharmacology for Natural Products, Kunming Medical University, Yunnan 650500, China
[4] The Affiliated Changsha Central Hospital, Hengyang Medical School, University of South China, Changsha 410004, China
* Correspondence: xyk@xtbg.ac.cn

Contents

Tables .... **Error! Bookmark not defined.**
Table S1. NO inhibitory effects of compounds **1~7**. 4
Table S2. Cytotoxic activity of compounds **1~7** (IC_{50}, μM). 4
Figures 5
Figure S1. ^{1}H NMR (500 MHz, $CDCl_3$) spectrum of **1**. 5
Figure S2. ^{13}C NMR (125 MHz, $CDCl_3$) spectrum of **1**. 5
Figure S3. HSQC (500 MHz, $CDCl_3$) spectrum of **1**. 6
Figure S4. HMBC (500 MHz, $CDCl_3$) spectrum of **1**. 6
Figure S5. ^{1}H-^{1}H COSY (500 MHz, $CDCl_3$) spectrum of **1**. 7
Figure S6. NOESY (500 MHz, $CDCl_3$) spectrum of **1**. 7
Figure S7. HR-ESI-MS spectrum of **1**. 8
Figure S8. ^{1}H NMR (500 MHz, $CDCl_3$) spectrum of **2**. 9
Figure S9. ^{13}C NMR (125 MHz, $CDCl_3$) spectrum of **2**. 9
Figure S10. HSQC (500 MHz, $CDCl_3$) spectrum of **2**. 10
Figure S11. HMBC (500 MHz, $CDCl_3$) spectrum of **2**. 10
Figure S12. ^{1}H-^{1}H COSY (500 MHz, $CDCl_3$) spectrum of **2**. 11
Figure S13. NOESY (500 MHz, $CDCl_3$) spectrum of **2**. 11
Figure S14. HR-ESI-MS spectrum of **2**. 12
Figure S15. ^{1}H NMR (500 MHz, $CDCl_3$) spectrum of **3**. 13
Figure S16. ^{13}C NMR (125 MHz, $CDCl_3$) spectrum of **3**. 13
Figure S17. HSQC (500 MHz, $CDCl_3$) spectrum of **3**. 14
Figure S18. HMBC (500 MHz, $CDCl_3$) spectrum of **3**. 14
Figure S19. ^{1}H-^{1}H COSY (500 MHz, $CDCl_3$) spectrum of **3**. 15
Figure S20. NOESY (500 MHz, $CDCl_3$) spectrum of **3**. 15
Figure S21. HR-ESI-MS spectrum of **3**. 16
Figure S22. ^{1}H NMR (500 MHz, $CDCl_3$) spectrum of **4**. 17
Figure S23. ^{13}C NMR (125 MHz, $CDCl_3$) spectrum of **4**. 17
Figure S24. HSQC (500 MHz, $CDCl_3$) spectrum of **4**. 18
Figure S25. HMBC (500 MHz, $CDCl_3$) spectrum of **4**. 18
Figure S26. ^{1}H-^{1}H COSY (500 MHz, $CDCl_3$) spectrum of **4**. 19
Figure S27. NOESY (500 MHz, $CDCl_3$) spectrum of **4**. 19
Figure S28. HR-ESI-MS spectrum of **4**. 20
Figure S29. ^{1}H NMR (500 MHz, $CDCl_3$) spectrum of **5**. 21
Figure S30. ^{13}C NMR (125 MHz, $CDCl_3$) spectrum of **5**. 21
Figure S31. HSQC (500 MHz, $CDCl_3$) spectrum of **5**. 22
Figure S32. HMBC (500 MHz, $CDCl_3$) spectrum of **5**. 22
Figure S33. ^{1}H-^{1}H COSY (500 MHz, $CDCl_3$) spectrum of **5**. 23
Figure S34. NOESY (500 MHz, $CDCl_3$) spectrum of **5**. 23
Figure S35. HR-ESI-MS spectrum of **5**. 24
Figure S36. ^{1}H NMR (500 MHz, $CDCl_3$) spectrum of **6**. 25
Figure S37. ^{13}C NMR (125 MHz, $CDCl_3$) spectrum of **6**. 25

Figure S38. HSQC (500 MHz, $CDCl_3$) spectrum of **6**. 26
Figure S39. HMBC (500 MHz, $CDCl_3$) spectrum of **6**. 26
Figure S40. ^1H-^1H COSY (500 MHz, $CDCl_3$) spectrum of **6**. 27
Figure S41. NOESY(500 MHz, $CDCl_3$) spectrum of **6**. 27
Figure S42. HR-ESI-MS spectrum of **6**. 28
Figure S43. ^1H NMR (500 MHz, $CDCl_3$) spectrum of **7**. 29
Figure S44. ^{13}C NMR (125 MHz, $CDCl_3$) spectrum of **7**. 29
Figure S45. HSQC (500 MHz, $CDCl_3$) spectrum of **7**. 30
Figure S46. HMBC (500 MHz, $CDCl_3$) spectrum of **7**. 30
Figure S47. ^1H-^1H COSY (500 MHz, $CDCl_3$) spectrum of **7**. 31
Figure S48. NOESY (500 MHz, $CDCl_3$) spectrum of **7**. 31
Figure S49. HR-ESI-MS spectrum of **7**. 32

Tables

Table S1. NO inhibitory effects of compounds **1**~**7**.

Compound	Concentration (μM)	NO Inhibition Rate (%)
**1**	50	-28.60±2.84
**2**	50	-19.03±3.52
**3**	50	-9.29±2.36
**4**	50	-9.18±2.25
**5**	50	41.62±0.95
**6**	50	4.62±2.53
**7**	50	10.34±2.17
L-NMMA[a]	50	54.94±1.43

[a] Positive control.

Table S2. Cytotoxic activity of compounds **1**~**7** (IC_{50}, μM).

Compound	HL-60	SMMC-7721	A-549	MCF-7	SW480
**1**	>40	>40	>40	>40	>40
**2**	>40	>40	>40	>40	>40
**3**	>40	>40	>40	>40	>40
**4**	>40	>40	>40	>40	>40
**5**	>40	>40	>40	>40	>40
**6**	>40	>40	>40	>40	>40
**7**	>40	>40	>40	>40	>40
Cisplatin[a]	2.75	4.78	3.56	16.20	10.69

[a] Positive control.

Figures

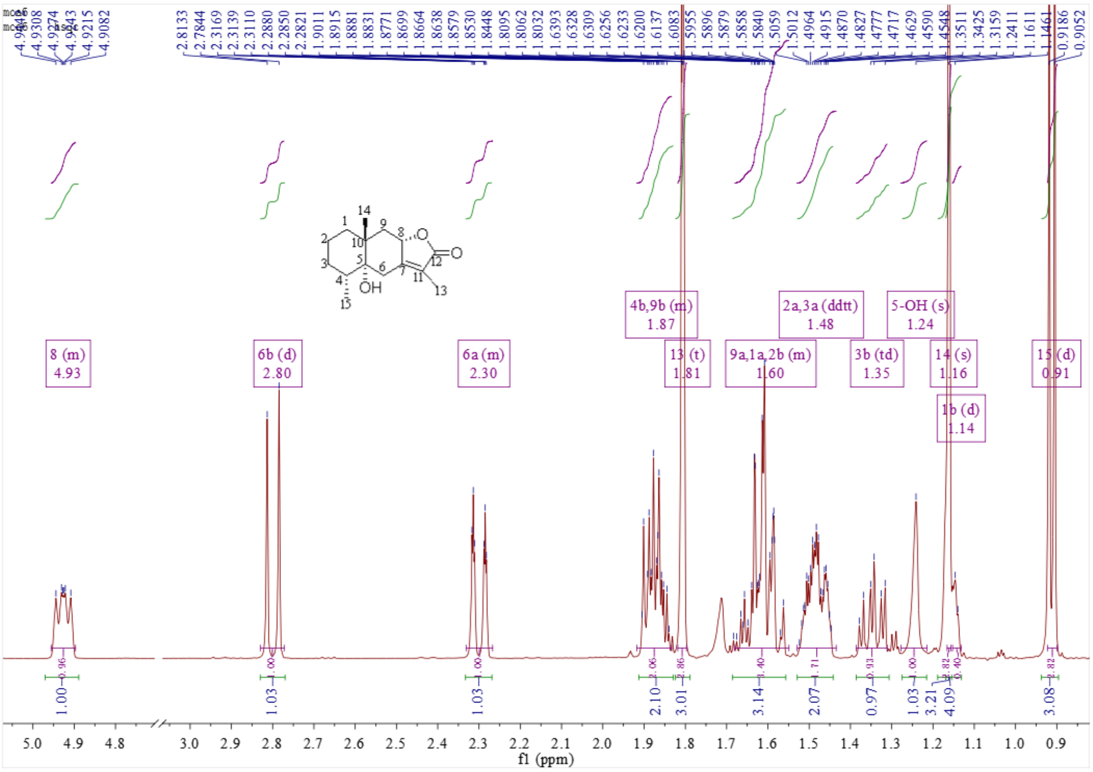

Figure S1. ^{1}H NMR (500 MHz, $CDCl_3$) spectrum of **1**.

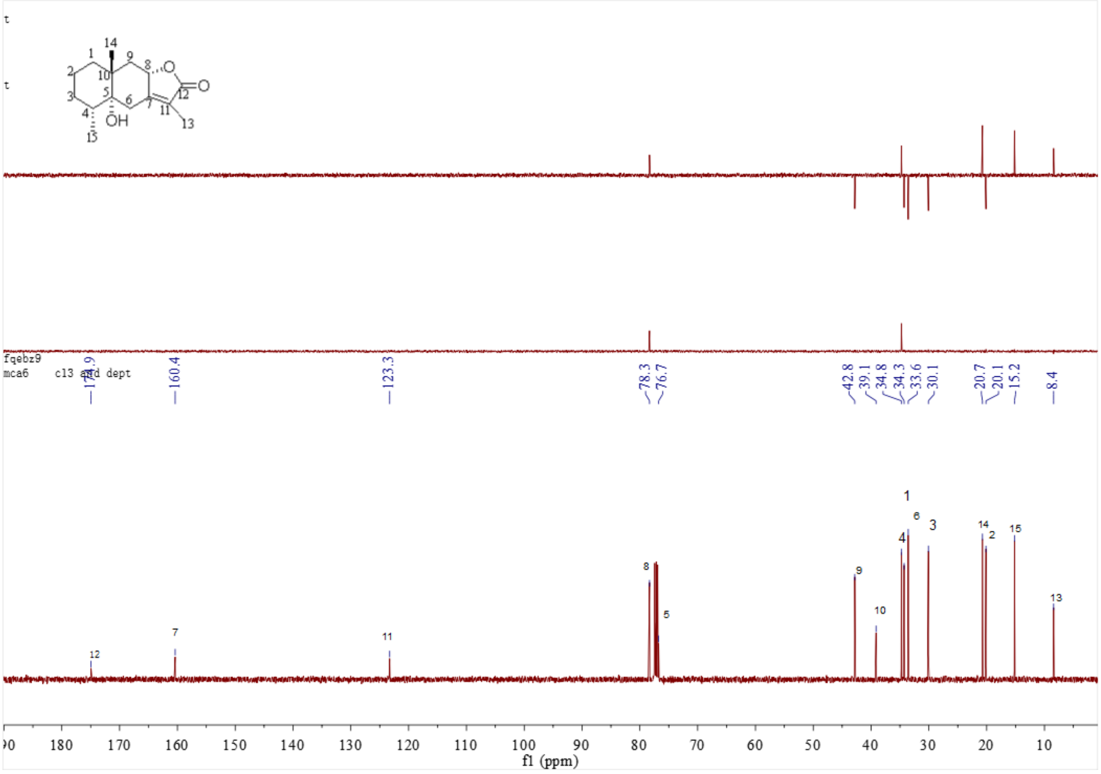

Figure S2. ^{13}C NMR (125 MHz, $CDCl_3$) spectrum of **1**.

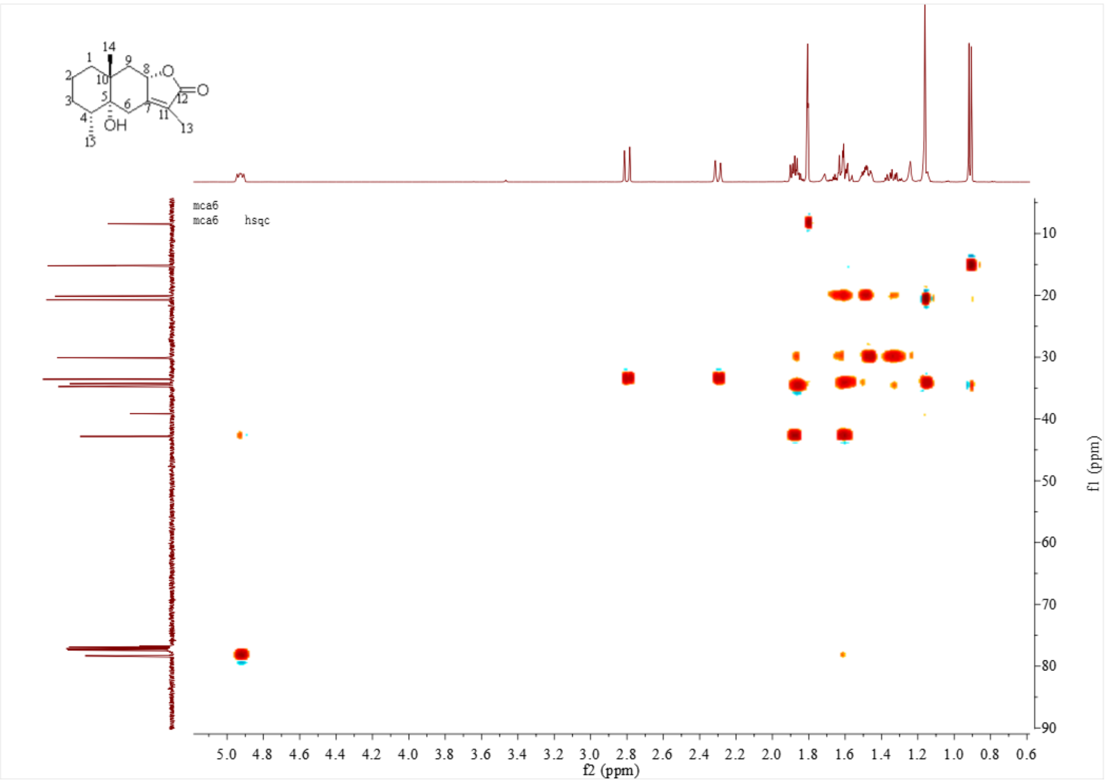

Figure S3. HSQC (500 MHz, CDCl3) spectrum of **1**.

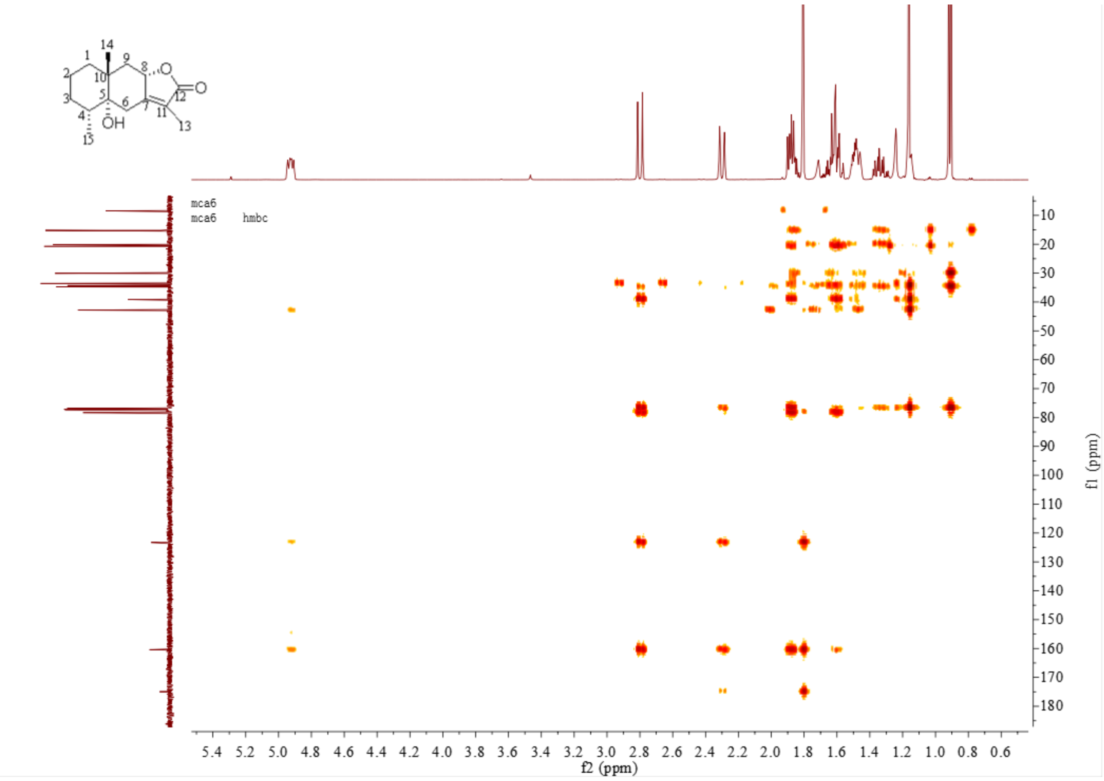

Figure S4. HMBC (500 MHz, CDCl3) spectrum of **1**.

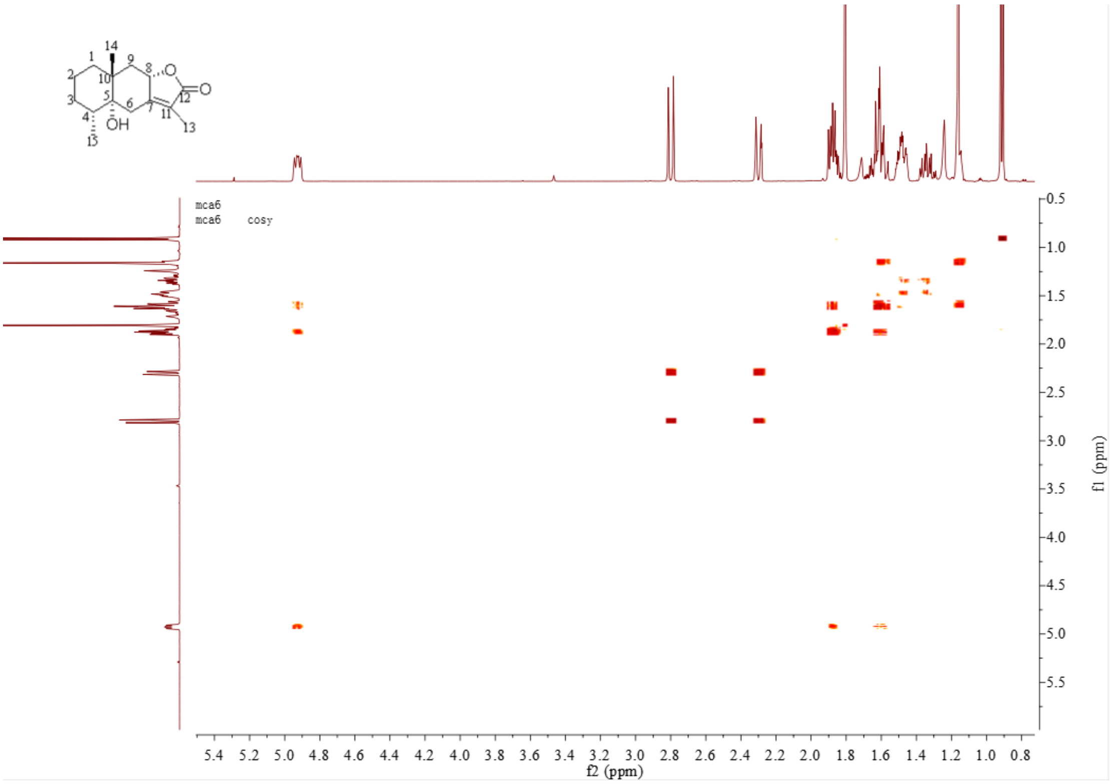

Figure S5. ^{1}H-^{1}H COSY (500 MHz, $CDCl_3$) spectrum of **1**.

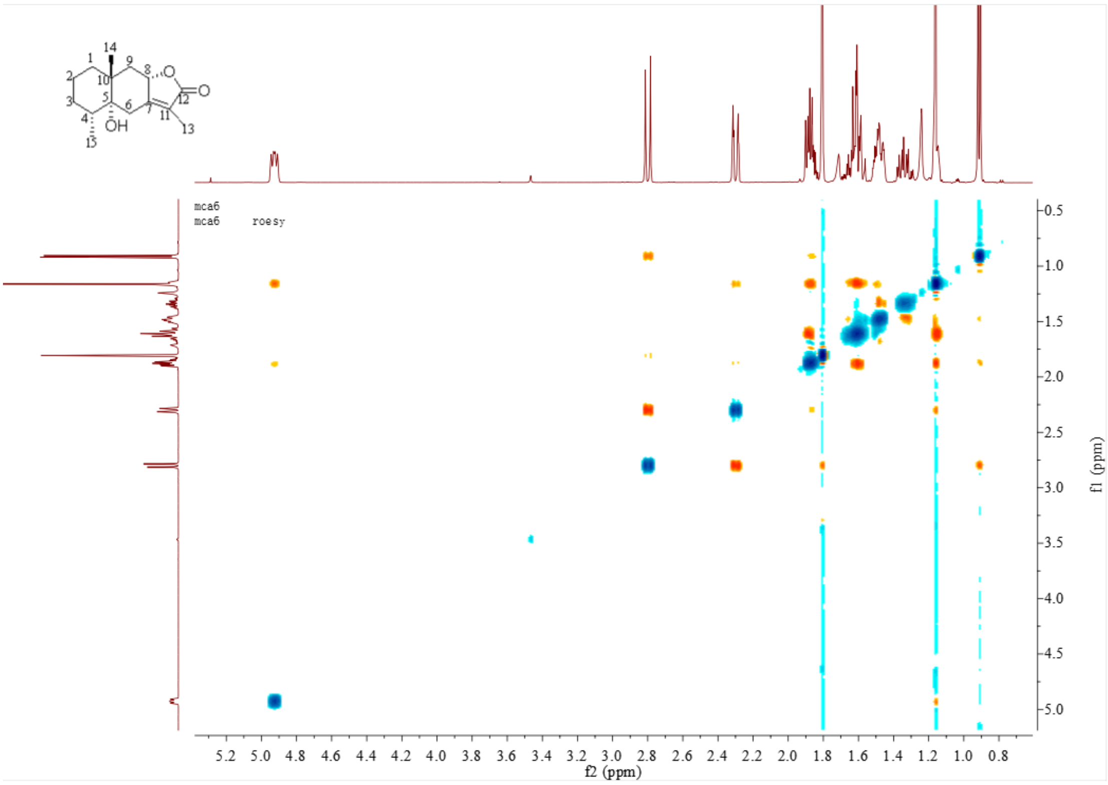

Figure S6. NOESY (500 MHz, $CDCl_3$) spectrum of **1**.

Formula Predictor Report - MCA-6.lcd Page 1 of 1

Data File: E:\DATA\2021\0714\MCA-6.lcd

Elmt	Val.	Min	Max	Elmt	Val.	Min	Max	Elmt	Val.	Min	Max	Elmt	Val.	Min	Max	Use Adduct
H	1	10	150	O	2	0	30	P	3	0	0	Se	2	0	0	H
2H	1	0	0	F	1	0	0	S	2	0	0	Br	1	0	0	Na
B	3	0	0	Na	1	0	0	Cl	1	0	0	Pd	2	0	0	
C	4	10	150	Mg	2	0	0	Co	2	0	0	Ag	1	0	0	
N	3	0	10	Si	4	0	0	Cu	2	0	0	I	3	0	0	

Error Margin (ppm):	5	DBE Range:	not fixed	Electron Ions:	both
HC Ratio:	unlimited	Apply N Rule:	yes	Use MSn Info:	yes
Max Isotopes:	all	Isotope RI (%):	1.00	Isotope Res:	10000
MSn Iso RI (%):	75.00	MSn Logic Mode:	OR	Max Results:	20

Event#: 1 MS(E+) Ret. Time : 0.400 -> 0.467 Scan# : 61 -> 71

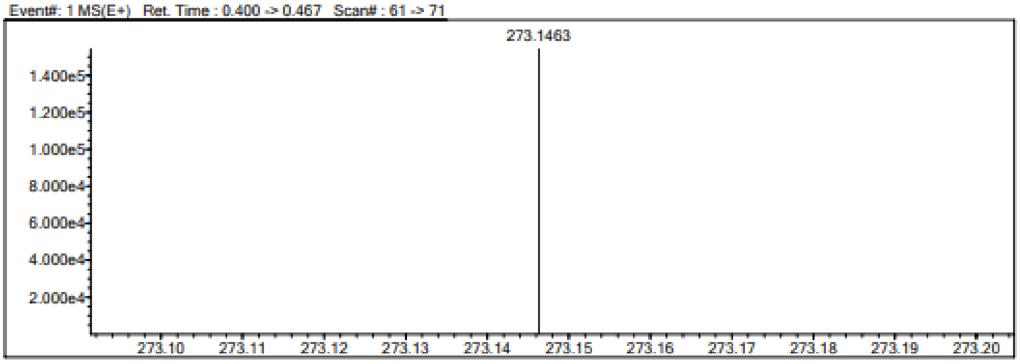

Measured region for 273.1463 m/z

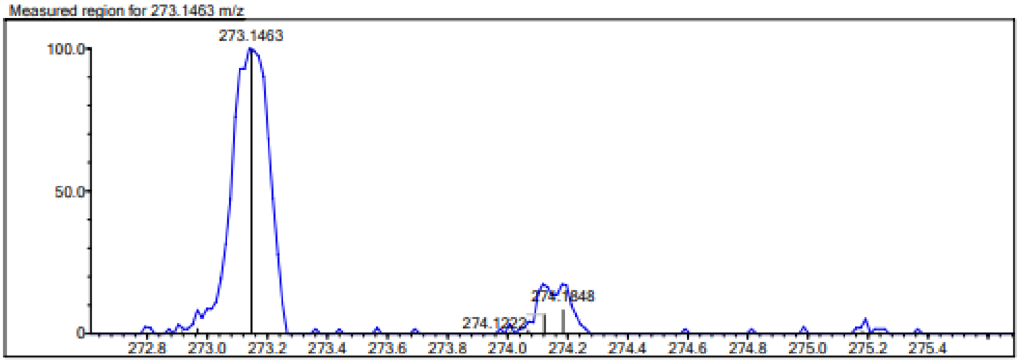

C15 H22 O3 [M+Na]+ : Predicted region for 273.1461 m/z

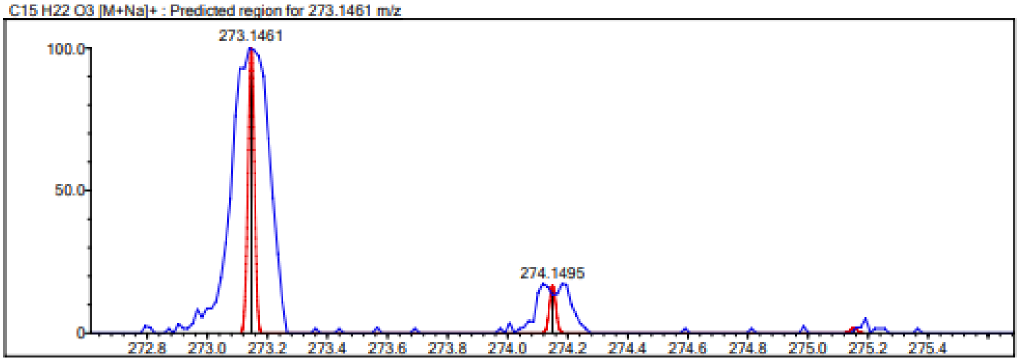

Formula (M)	Ion	Meas. m/z	Pred. m/z	Df. (mDa)	Df. (ppm)	DBE
C15 H22 O3	[M+Na]+	273.1463	273.1461	0.2	0.73	5.0

Figure S7. HR-ESI-MS spectrum of **1**.

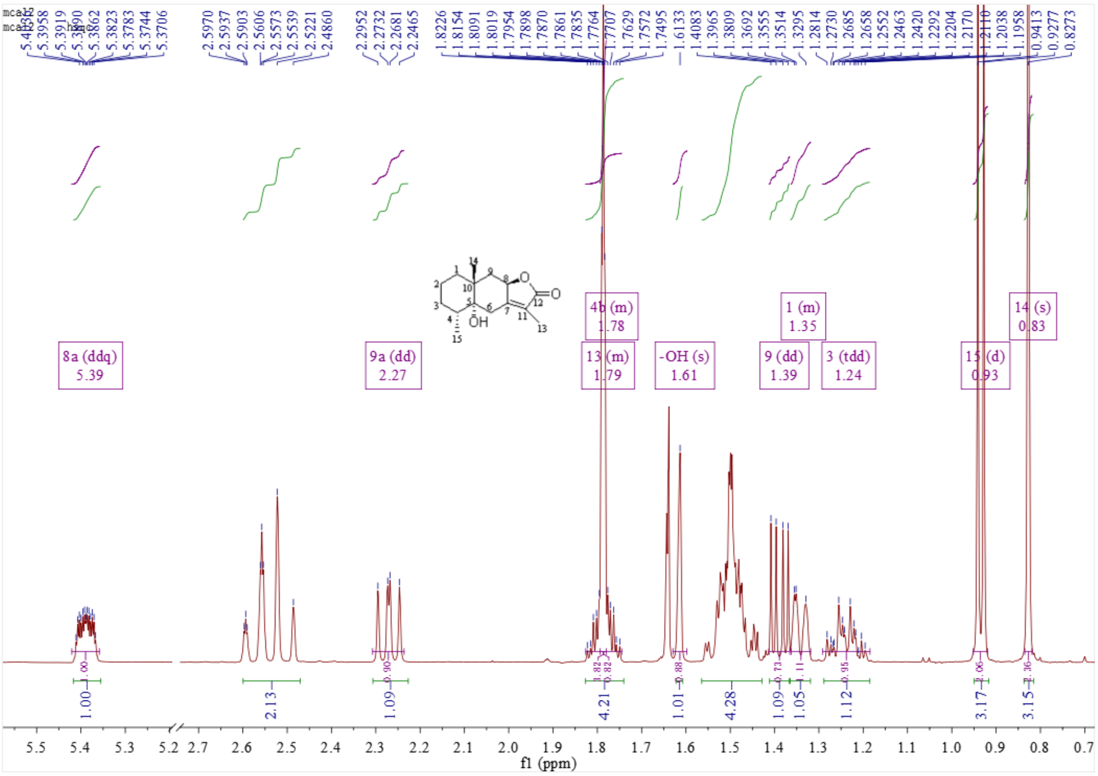

Figure S8. ^{1}H NMR (500 MHz, $CDCl_3$) spectrum of **2**.

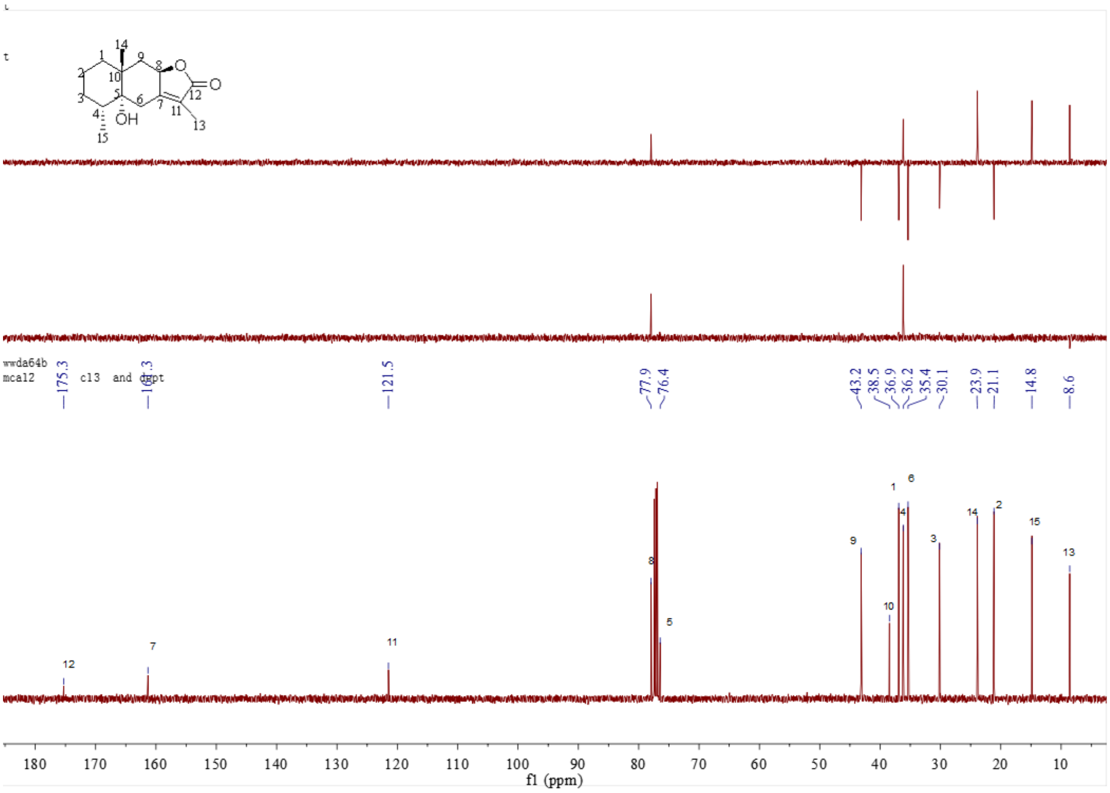

Figure S9. ^{13}C NMR (125 MHz, $CDCl_3$) spectrum of **2**.

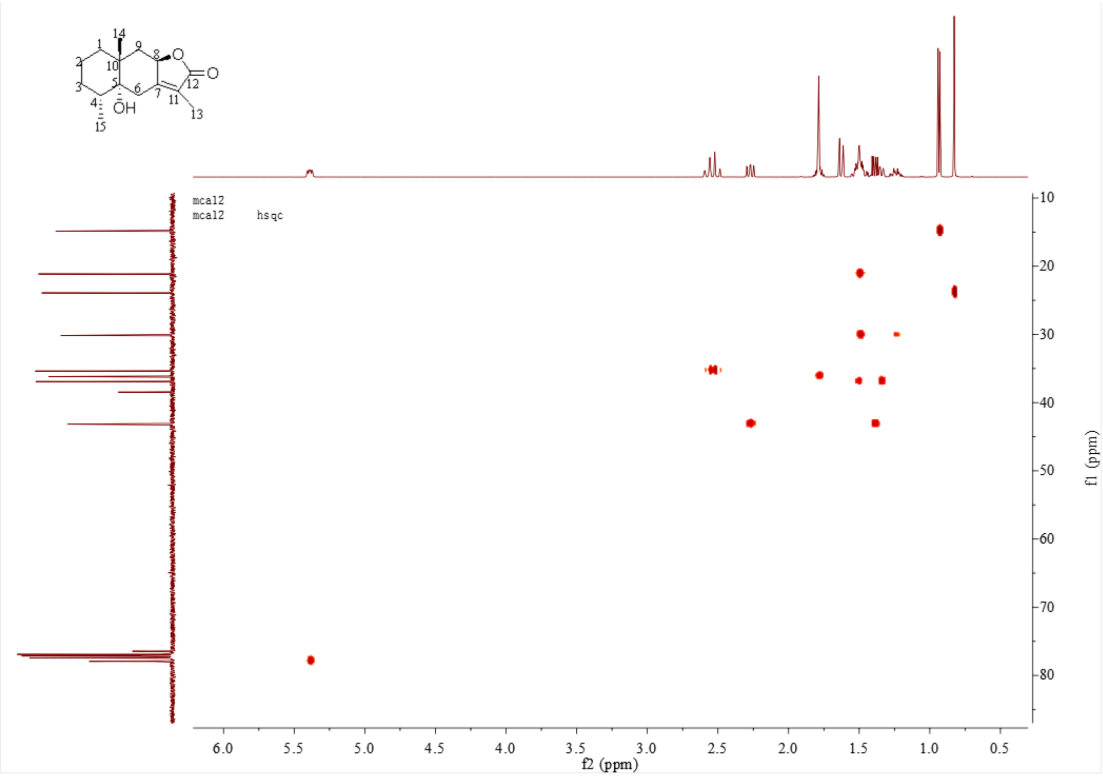

Figure S10. HSQC (500 MHz, CDCl3) spectrum of **2**.

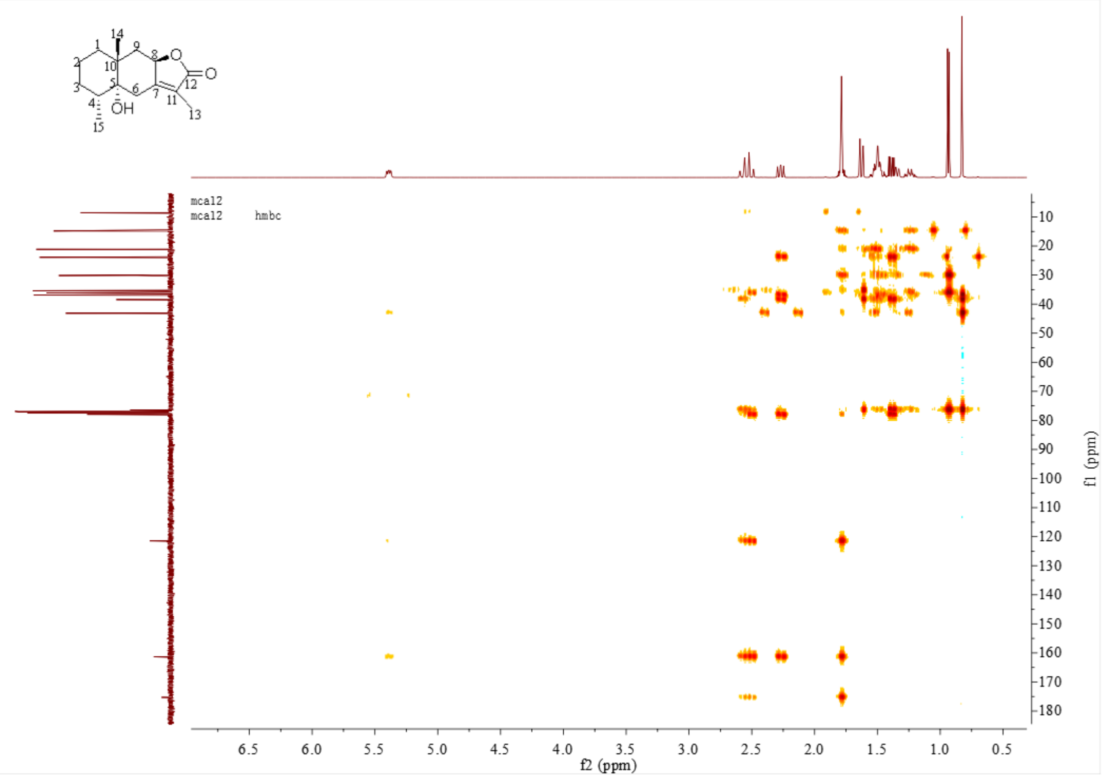

Figure S11. HMBC (500 MHz, CDCl3) spectrum of **2**.

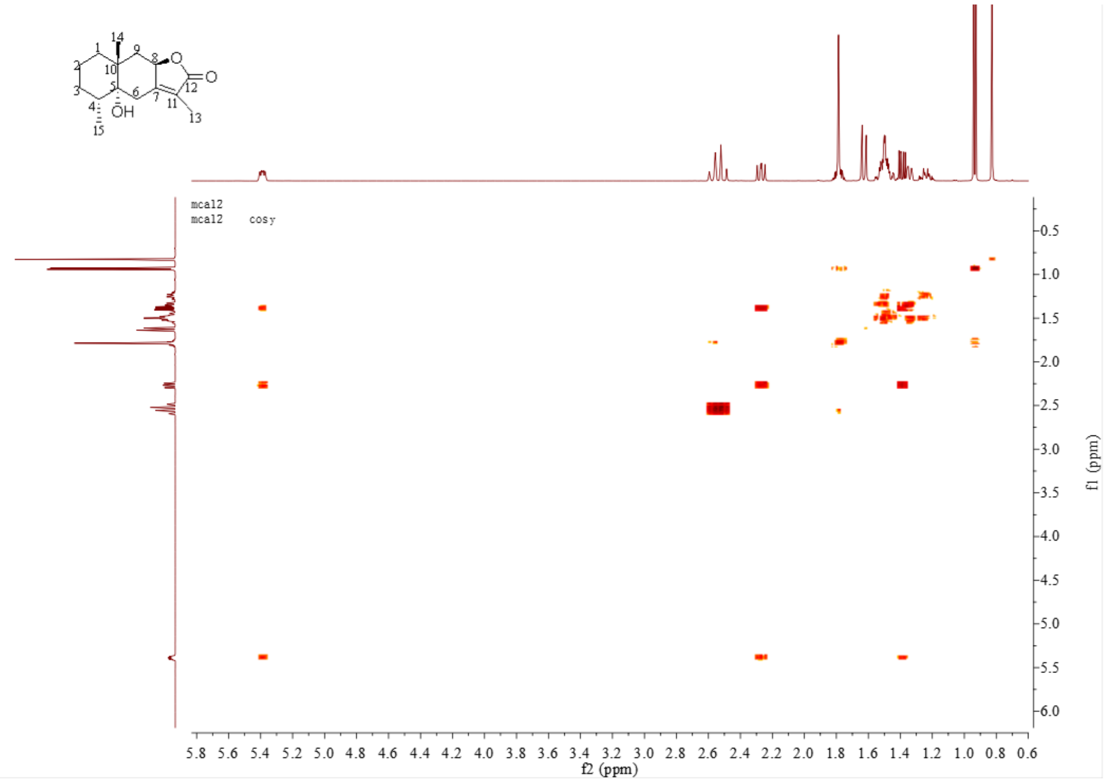

Figure S12. ^{1}H-^{1}H COSY (500 MHz, $CDCl_3$) spectrum of **2**.

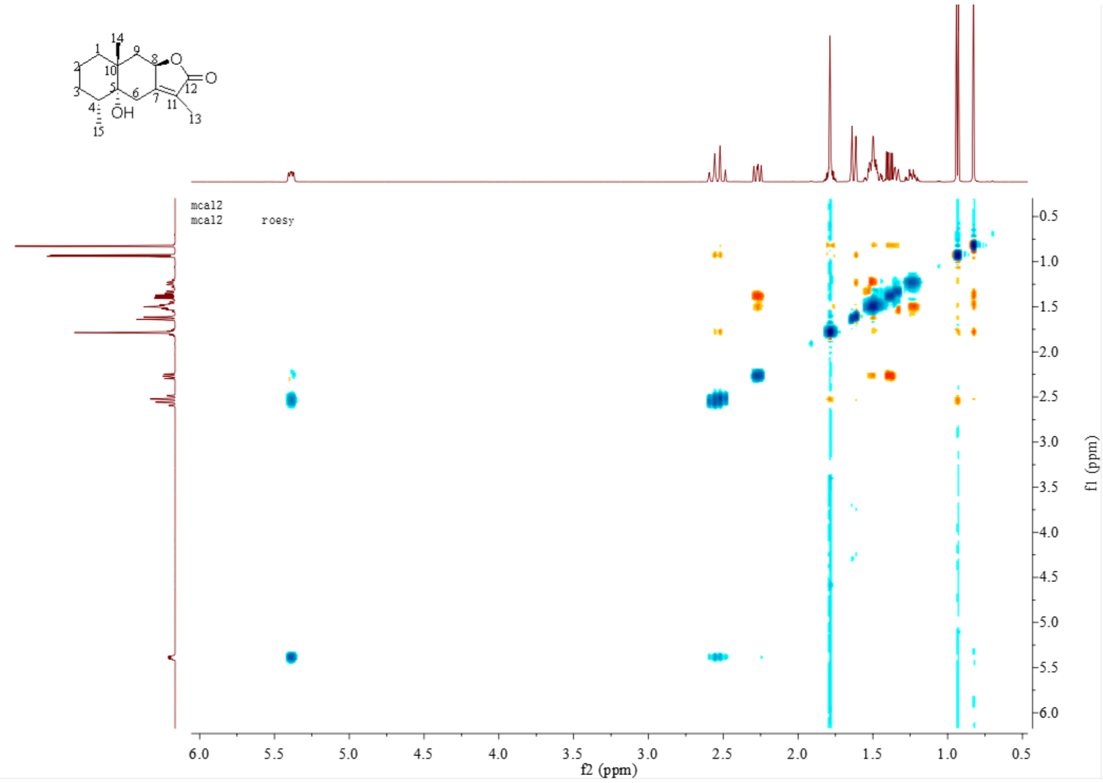

Figure S13. NOESY (500 MHz, $CDCl_3$) spectrum of **2**.

Formula Predictor Report - MCA-12.lcd Page 1 of 1

Data File: E:\DATA\2021\1020\MCA-12.lcd

Elmt	Val.	Min	Max	Elmt	Val.	Min	Max	Elmt	Val.	Min	Max	Elmt	Val.	Min	Max	Use Adduct
H	1	5	100	F	1	0	0	Cl	1	0	0	Ag	1	0	0	H
2H	1	0	0	Na	1	0	0	Co	2	0	0	I	3	0	0	HCOO
B	3	0	0	Mg	2	0	0	Cu	2	0	0	Ir	3	0	0	
C	4	5	50	Si	4	0	0	Se	2	0	0					
N	3	0	10	P	3	0	0	Br	1	0	0					
O	2	0	30	S	2	0	0	Pd	2	0	0					

Error Margin (ppm):	5	DBE Range:	not fixed	Electron Ions:	both
HC Ratio:	unlimited	Apply N Rule:	yes	Use MSn Info:	yes
Max Isotopes:	all	Isotope RI (%):	1.00	Isotope Res:	10000
MSn Iso RI (%):	75.00	MSn Logic Mode:	OR	Max Results:	20

Event#: 2 MS(E-) Ret. Time : 0.387 -> 0.653 Scan# : 60 -> 100

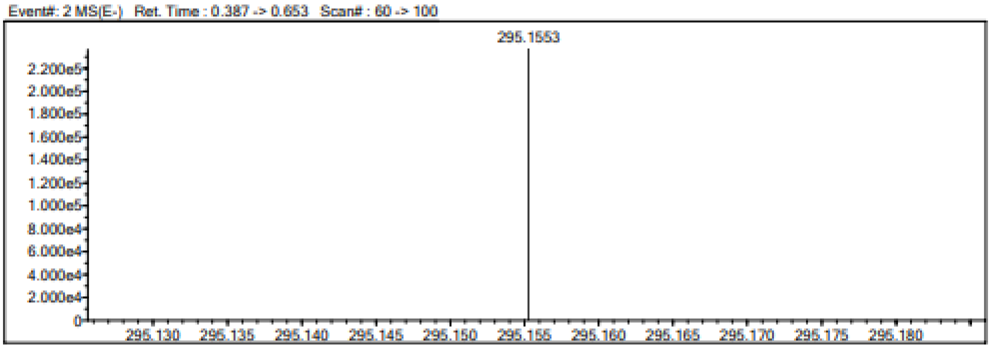

Measured region for 295.1553 m/z

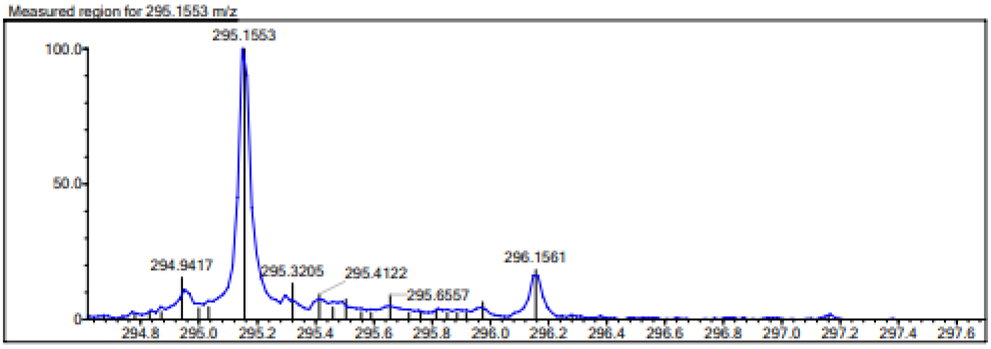

C15 H22 O3 [M+HCOO]- : Predicted region for 295.1551 m/z

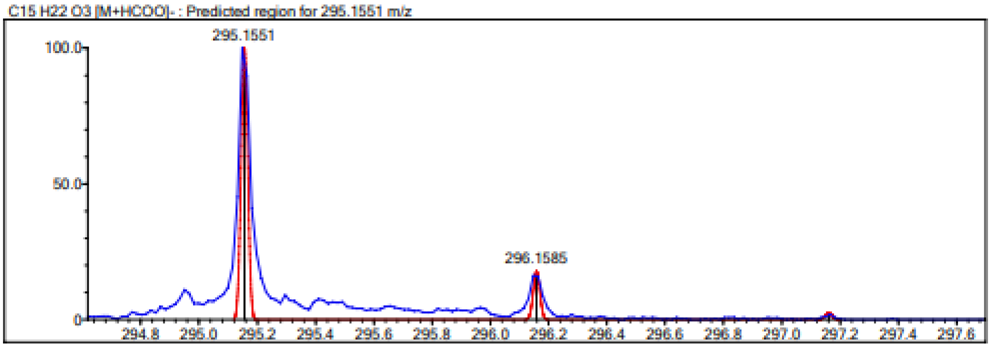

Formula (M)	Ion	Meas. m/z	Pred. m/z	Df. (mDa)	Df. (ppm)	DBE
C15 H22 O3	[M+HCOO]-	295.1553	295.1551	0.2	0.68	5.0

Figure S14. HR-ESI-MS spectrum of **2**.

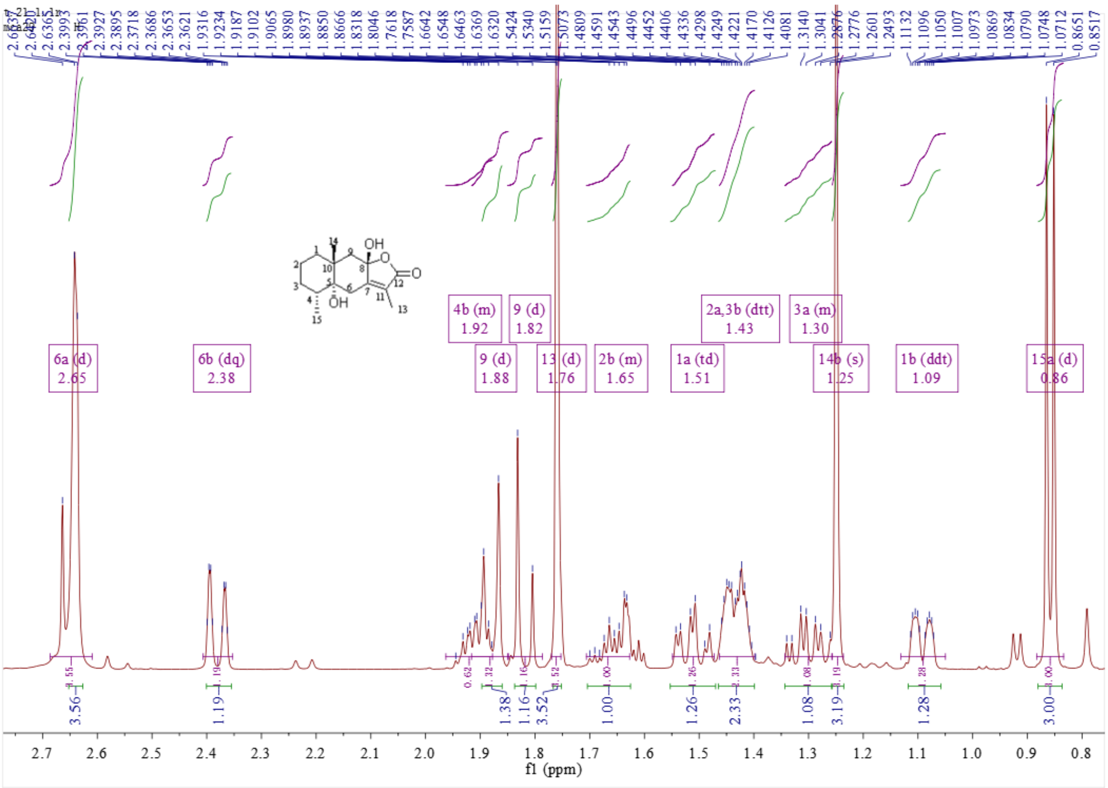

Figure S15. ^{1}H NMR (500 MHz, $CDCl_3$) spectrum of **3**.

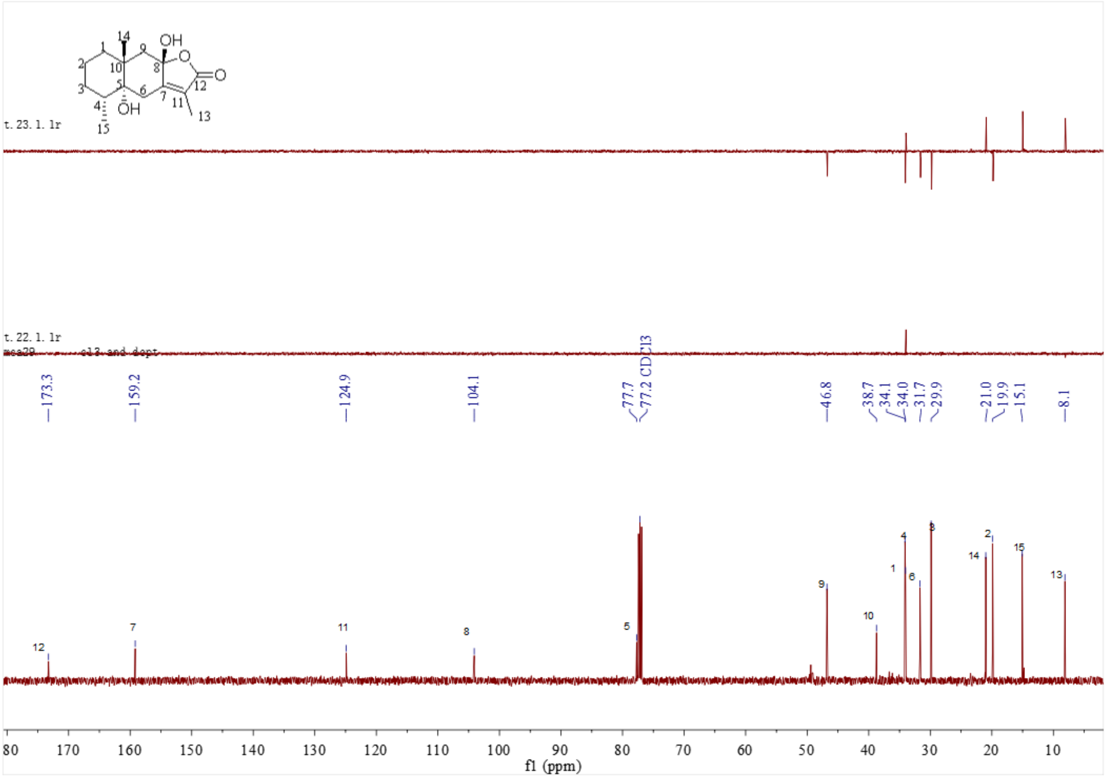

Figure S16. ^{13}C NMR (125 MHz, $CDCl_3$) spectrum of **3**.

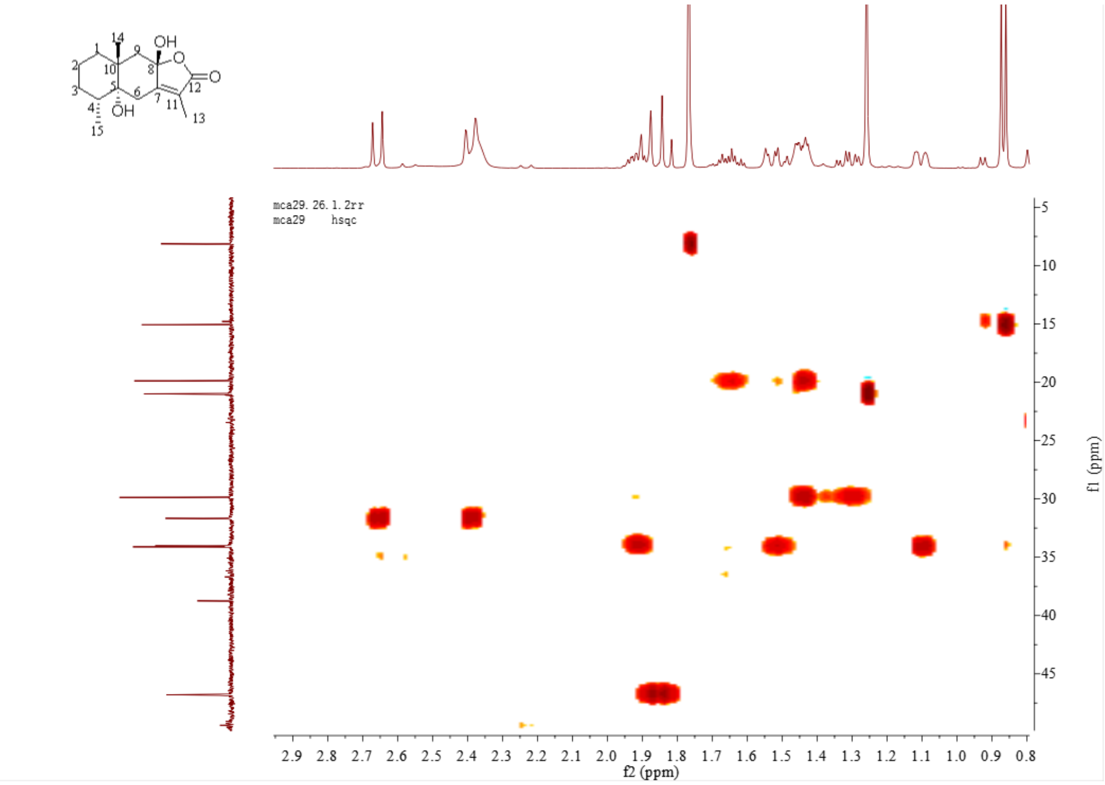

Figure S17. HSQC (500 MHz, CDCl3) spectrum of **3**.

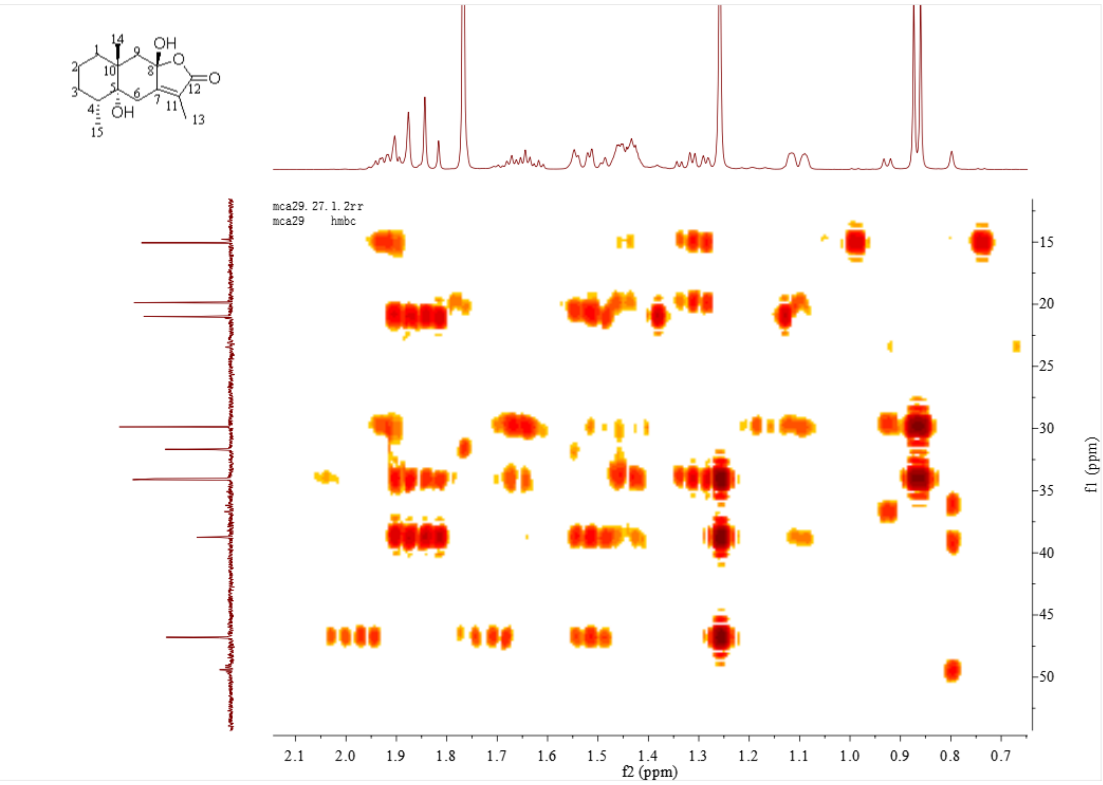

Figure S18. HMBC (500 MHz, CDCl3) spectrum of **3**.

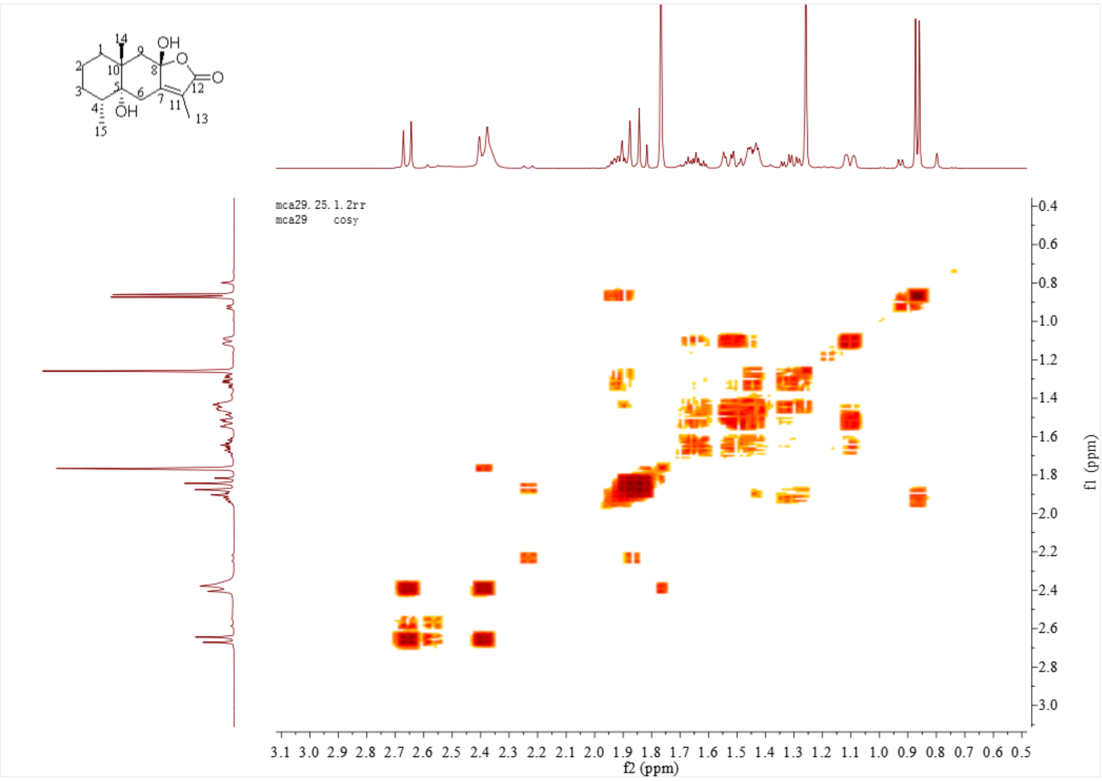

Figure S19. ^{1}H-^{1}H COSY (500 MHz, CDCl$_3$) spectrum of **3**.

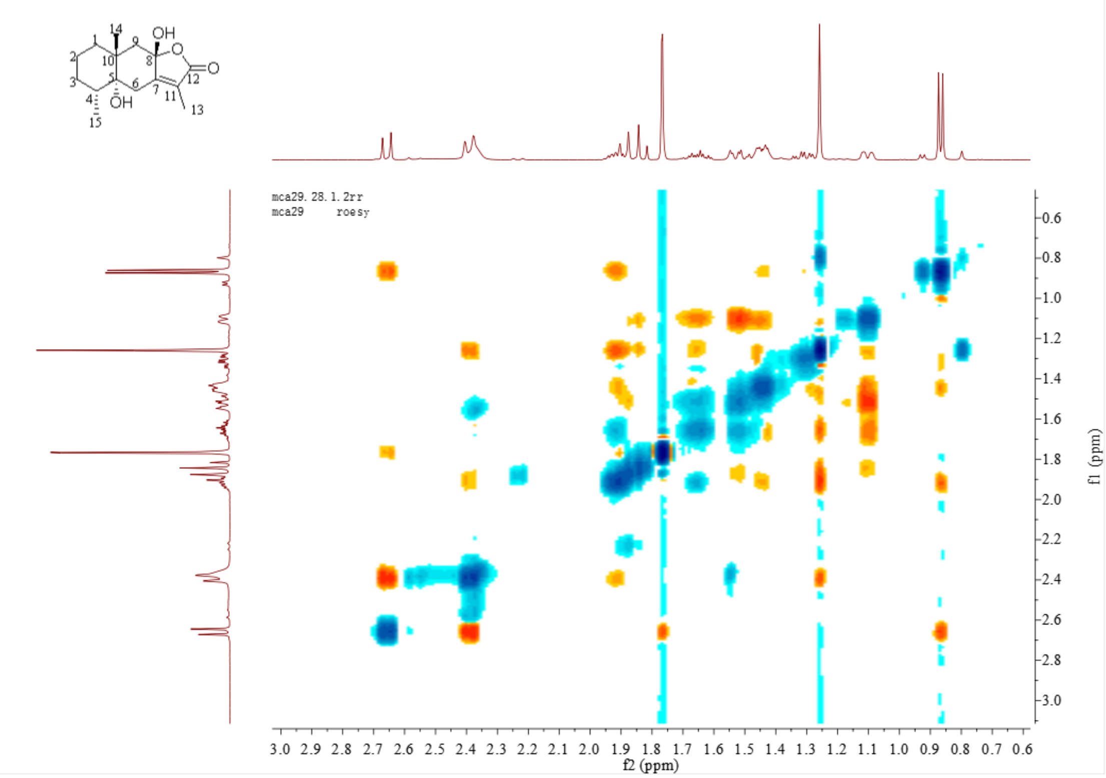

Figure S20. NOESY (500 MHz, CDCl$_3$) spectrum of **3**.

Formula Predictor Report - MCA-29.lcd Page 1 of 1

Data File: E:\DATA\2021\1020\MCA-29.lcd

Elmt	Val.	Min	Max	Elmt	Val.	Min	Max	Elmt	Val.	Min	Max	Elmt	Val.	Min	Max	Use Adduct
H	1	5	100	F	1	0	0	Cl	1	0	0	Ag	1	0	0	H
2H	1	0	0	Na	1	0	0	Co	2	0	0	I	3	0	0	HCOO
B	3	0	0	Mg	2	0	0	Cu	2	0	0	Ir	3	0	0	
C	4	5	50	Si	4	0	0	Se	2	0	0					
N	3	0	10	P	3	0	0	Br	1	0	0					
O	2	0	30	S	2	0	0	Pd	2	0	0					

Error Margin (ppm): 5
HC Ratio: unlimited
Max Isotopes: all
MSn Iso RI (%): 75.00

DBE Range: not fixed
Apply N Rule: yes
Isotope RI (%): 1.00
MSn Logic Mode: OR

Electron Ions: both
Use MSn Info: yes
Isotope Res: 10000
Max Results: 20

Event#: 2 MS(E-) Ret. Time : 0.387 -> 0.400 Scan# : 60 -> 62

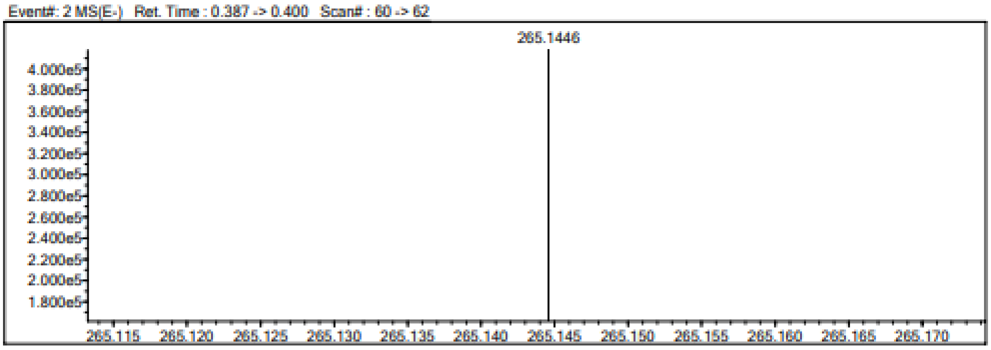

Measured region for 265.1446 m/z

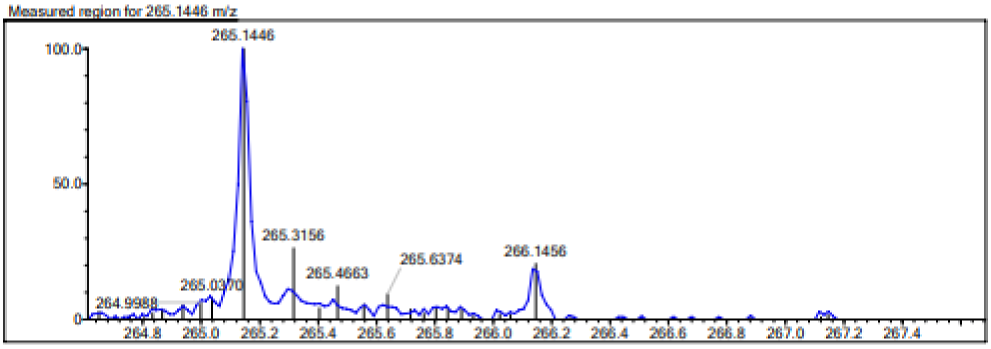

C15 H22 O4 [M-H]- : Predicted region for 265.1445 m/z

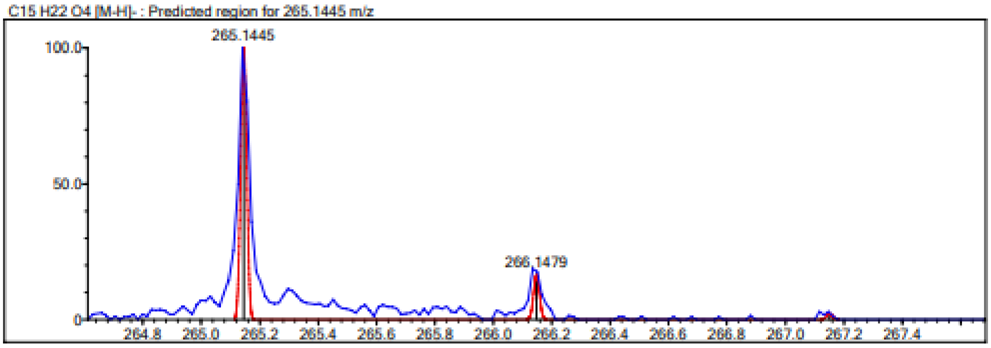

Formula (M)	Ion	Meas. m/z	Pred. m/z	Df. (mDa)	Df. (ppm)	DBE
C15 H22 O4	[M-H]-	265.1446	265.1445	0.1	0.38	5.0

Figure S21. HR-ESI-MS spectrum of **3**.

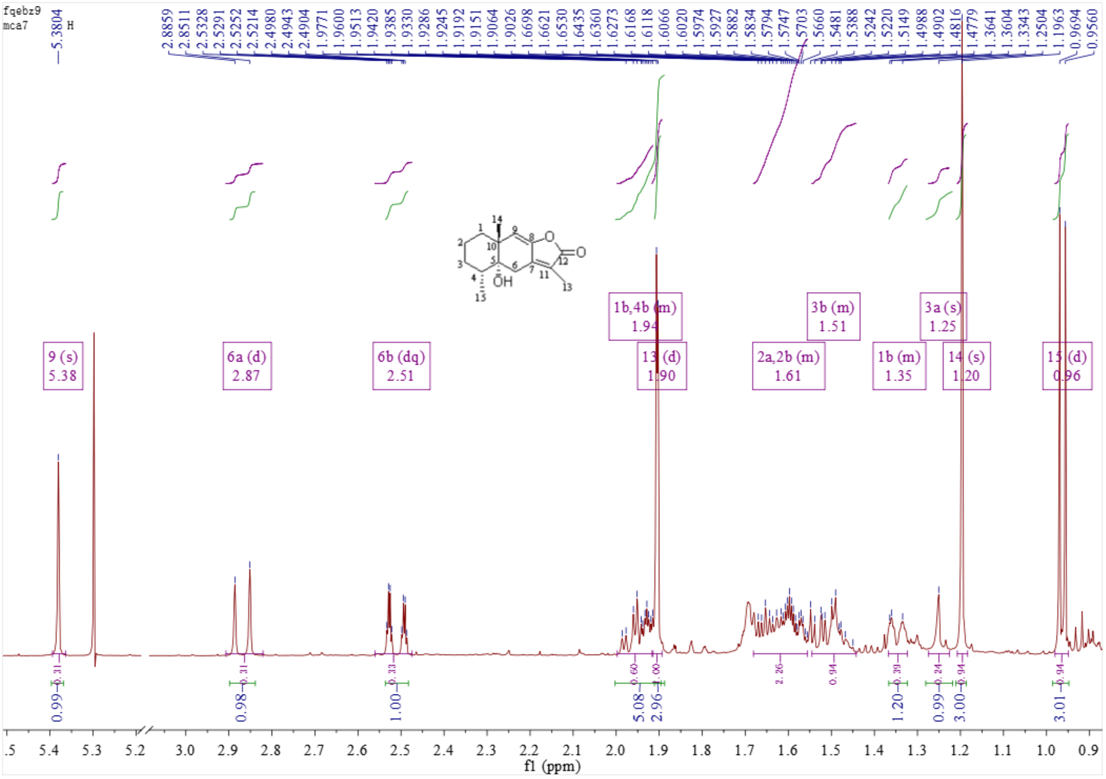

Figure S22. ^{1}H NMR (500 MHz, $CDCl_3$) spectrum of **4**.

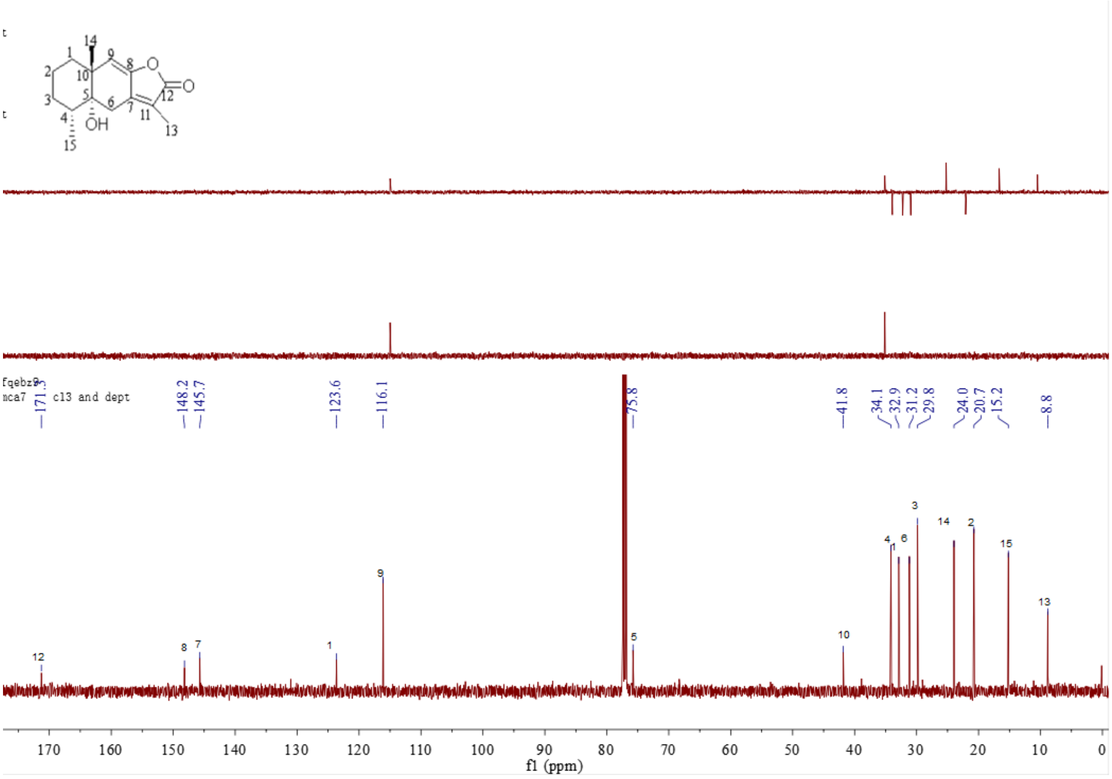

Figure S23. ^{13}C NMR (125 MHz, $CDCl_3$) spectrum of **4**.

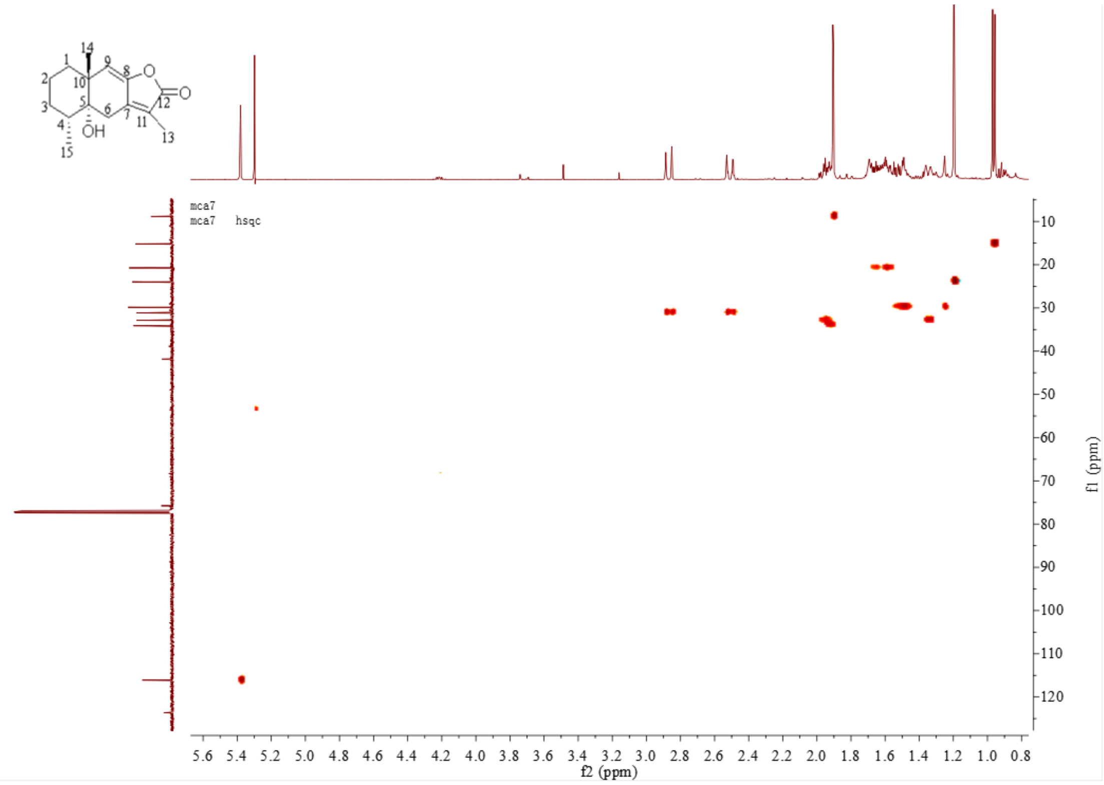

Figure S24. HSQC (500 MHz, CDCl3) spectrum of **4**.

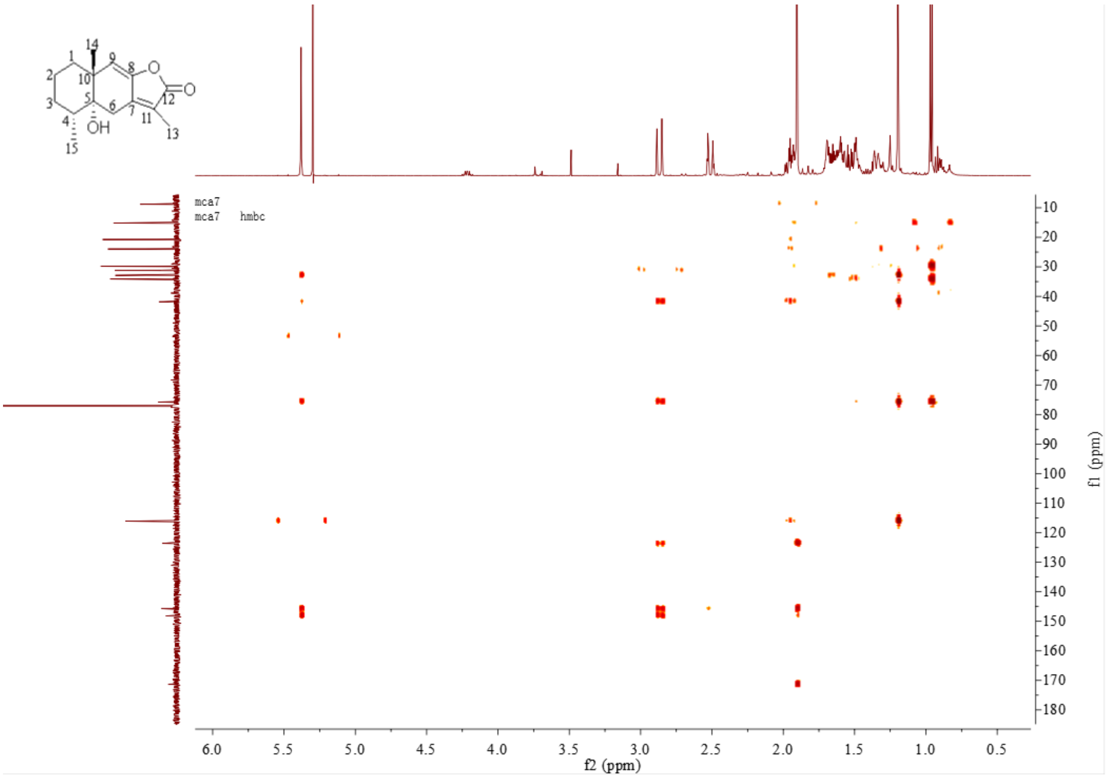

Figure S25. HMBC (500 MHz, CDCl3) spectrum of **4**.

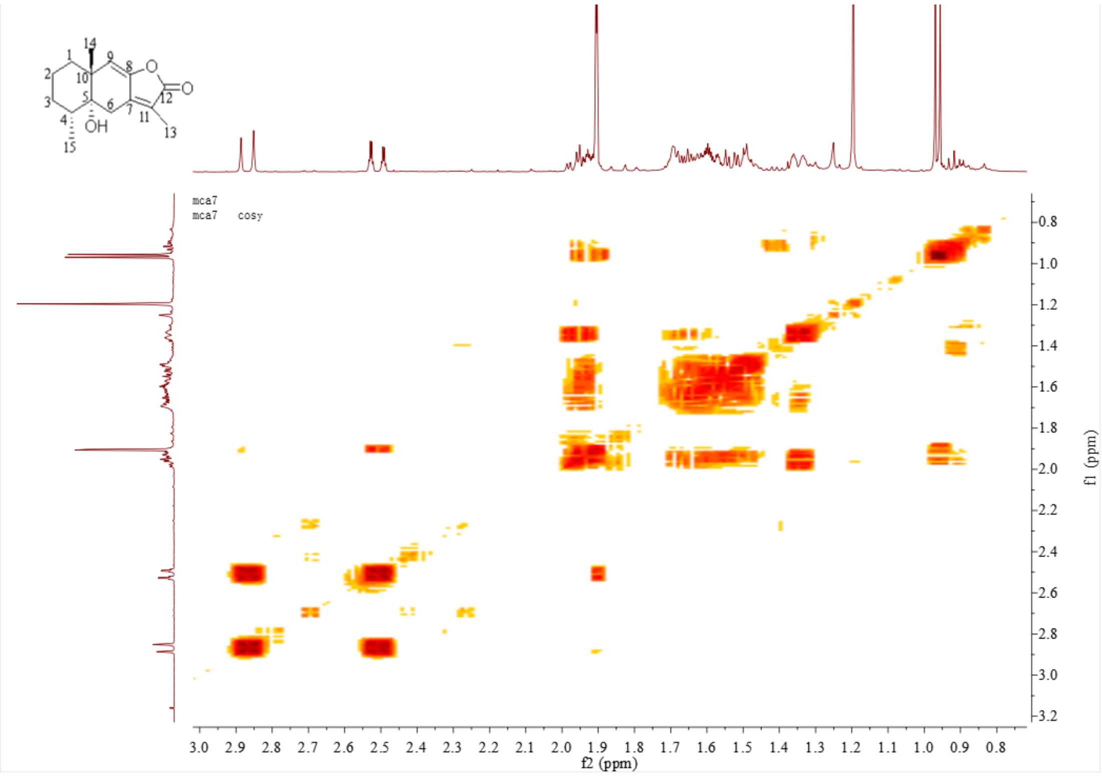

Figure S26. ^1H-^1H COSY (500 MHz, $CDCl_3$) spectrum of **4**.

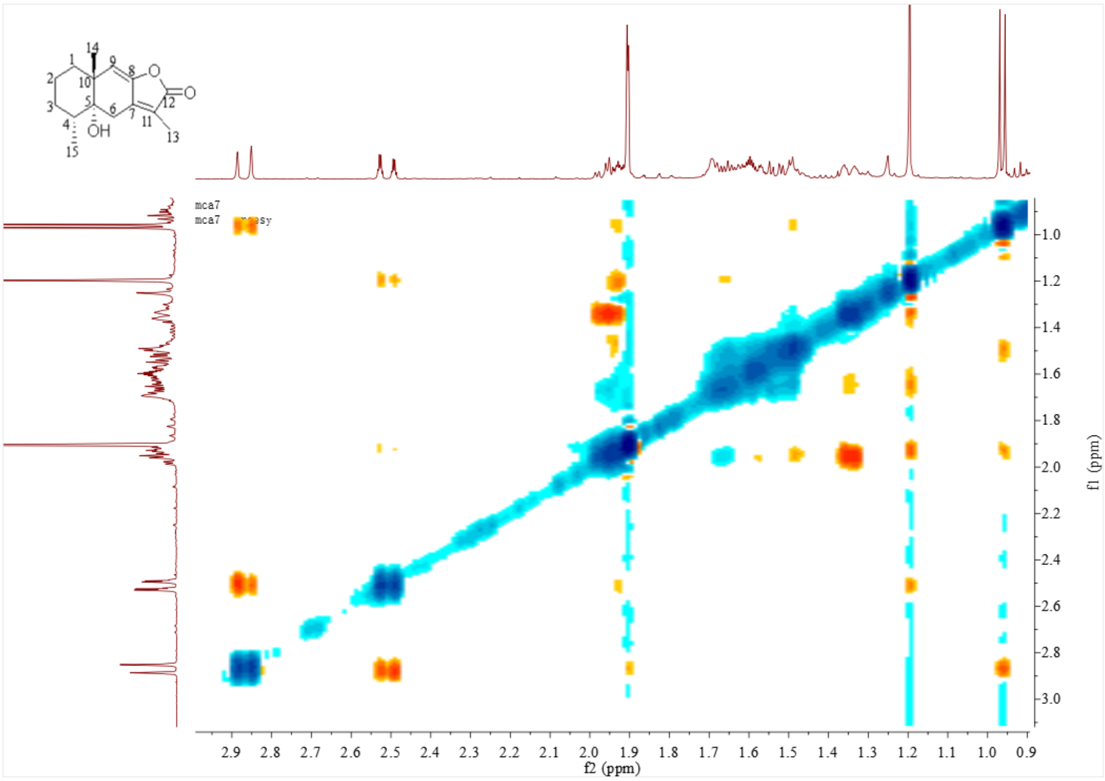

Figure S27. NOESY (500 MHz, $CDCl_3$) spectrum of **4**.

Formula Predictor Report - MCA-7.lcd Page 1 of 1

Data File: E:\DATA\2021\0714\MCA-7.lcd

Elmt	Val.	Min	Max	Elmt	Val.	Min	Max	Elmt	Val.	Min	Max	Elmt	Val.	Min	Max	Use Adduct
H	1	10	150	O	2	0	30	P	3	0	0	Se	2	0	0	H
2H	1	0	0	F	1	0	0	S	2	0	0	Br	1	0	0	Na
B	3	0	0	Na	1	0	0	Cl	1	0	0	Pd	2	0	0	
C	4	10	150	Mg	2	0	0	Co	2	0	0	Ag	1	0	0	
N	3	0	10	Si	4	0	0	Cu	2	0	0	I	3	0	0	

Error Margin (ppm): 5
HC Ratio: unlimited
Max Isotopes: all
MSn Iso RI (%): 75.00

DBE Range: not fixed
Apply N Rule: yes
Isotope RI (%): 1.00
MSn Logic Mode: OR

Electron Ions: both
Use MSn Info: yes
Isotope Res: 10000
Max Results: 20

Event#: 1 MS(E+) Ret. Time : 0.320 -> 1.000 Scan# : 49 -> 151

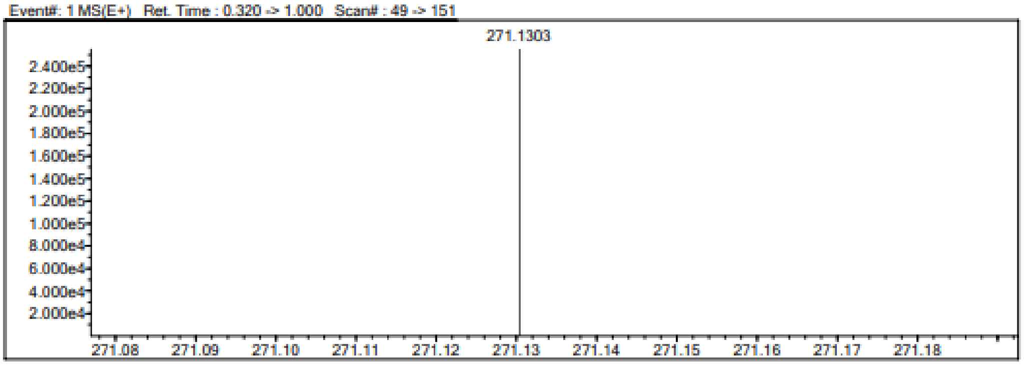

Measured region for 271.1303 m/z

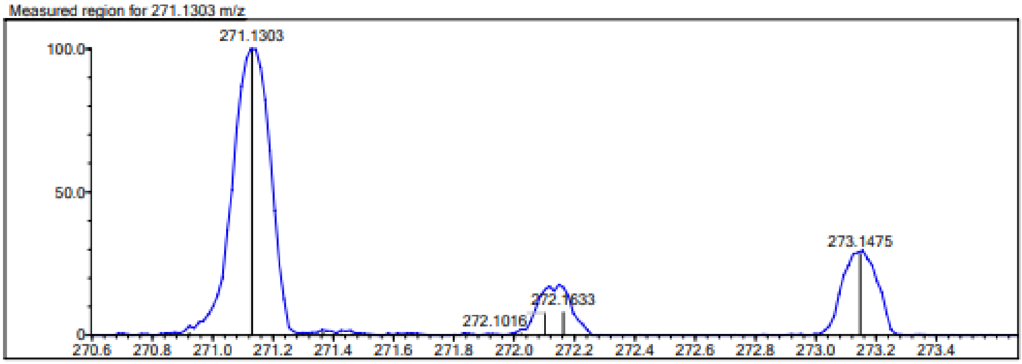

C15 H20 O3 [M+Na]+ : Predicted region for 271.1305 m/z

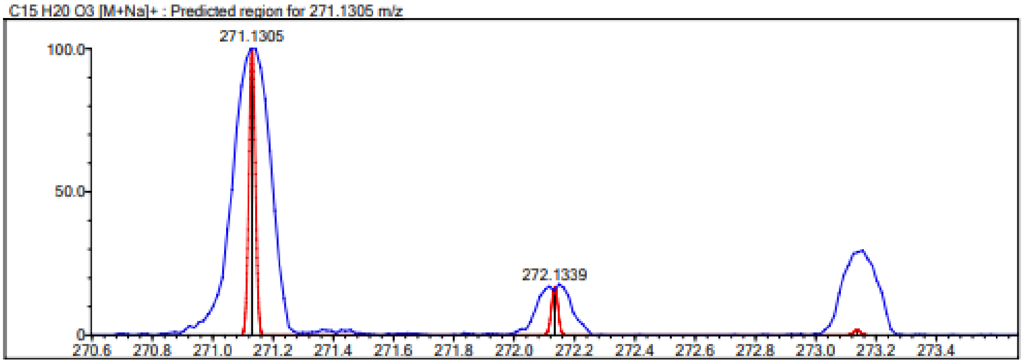

Formula (M)	Ion	Meas. m/z	Pred. m/z	Df. (mDa)	Df. (ppm)	DBE
C15 H20 O3	[M+Na]+	271.1303	271.1305	-0.2	-0.74	6.0

Figure S28. HR-ESI-MS spectrum of **4**.

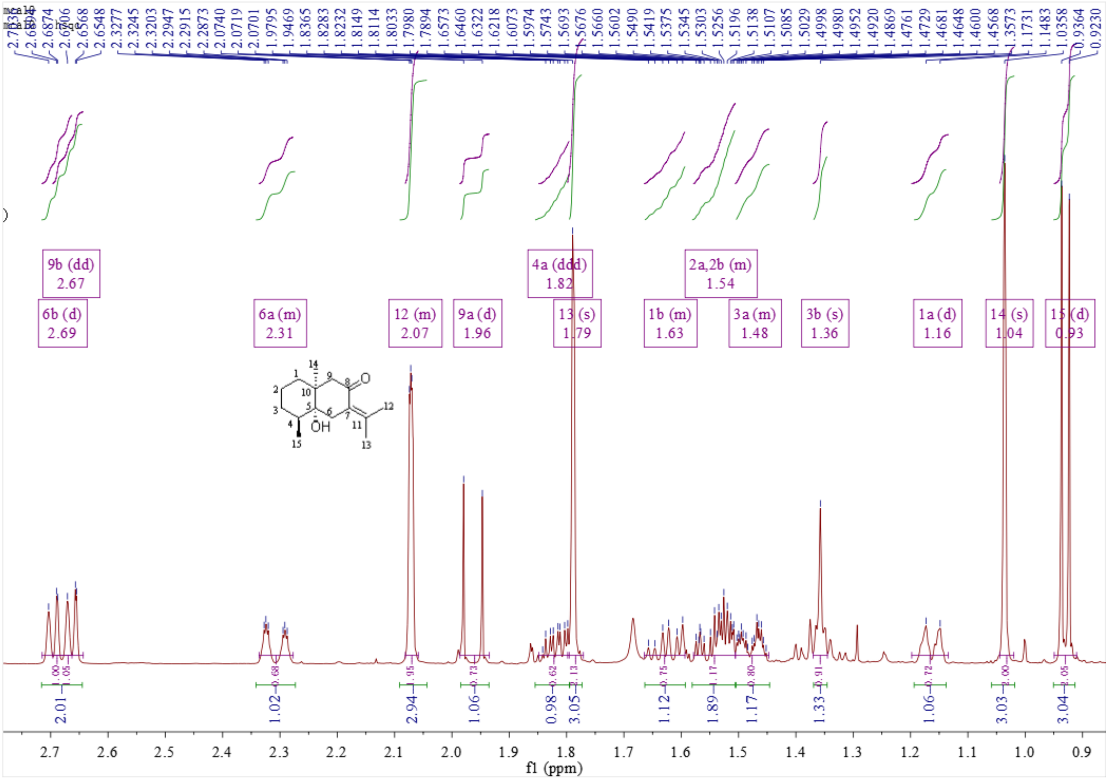

Figure S29. ^{1}H NMR (500 MHz, $CDCl_3$) spectrum of **5**.

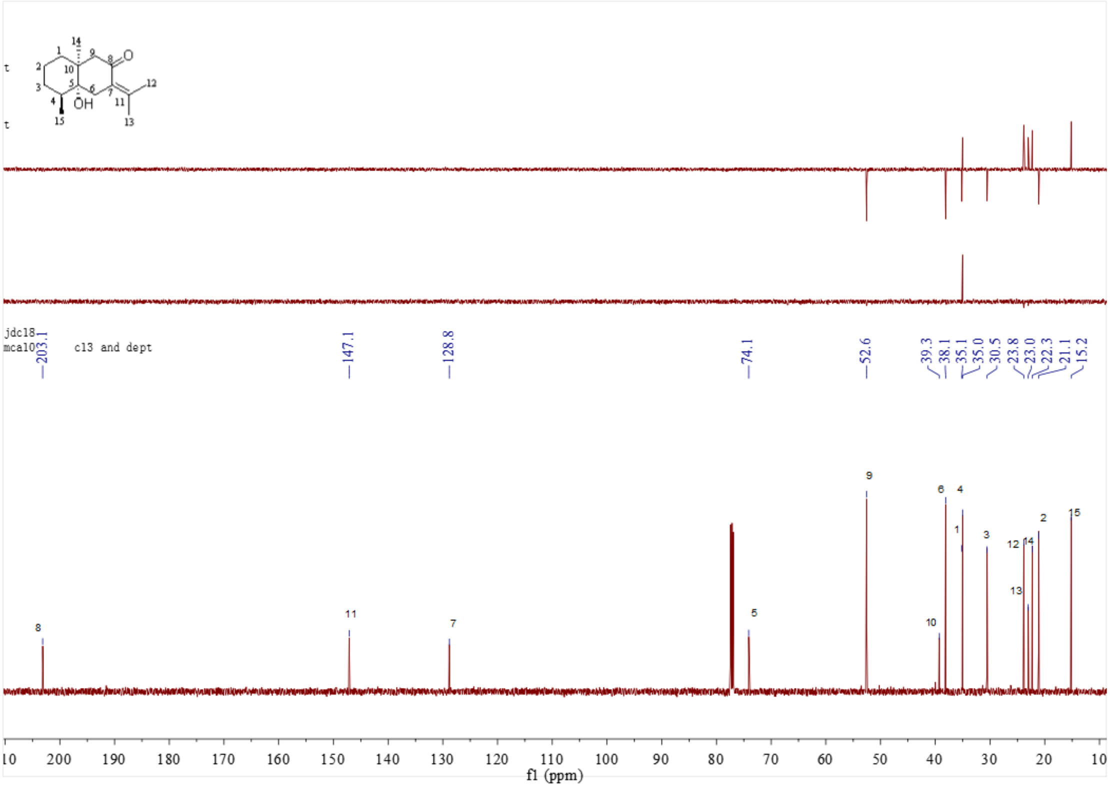

Figure S30. ^{13}C NMR (125 MHz, $CDCl_3$) spectrum of **5**.

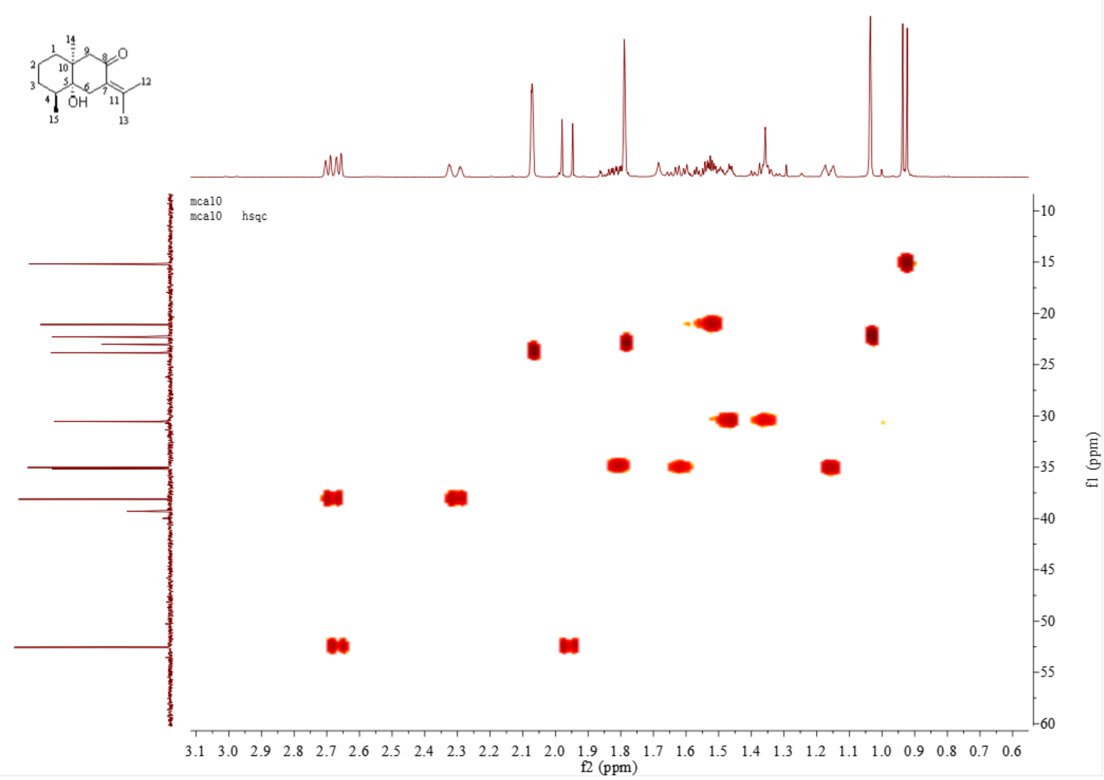

Figure S31. HSQC (500 MHz, $CDCl_3$) spectrum of **5**.

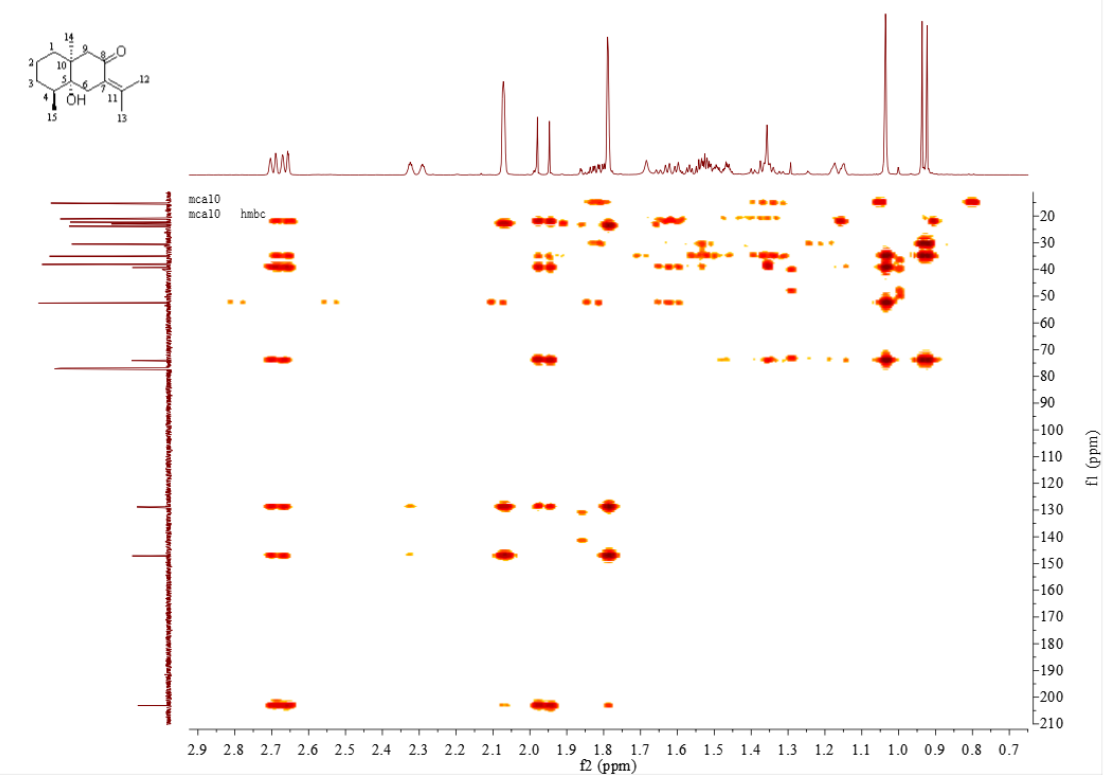

Figure S32. HMBC (500 MHz, $CDCl_3$) spectrum of **5**.

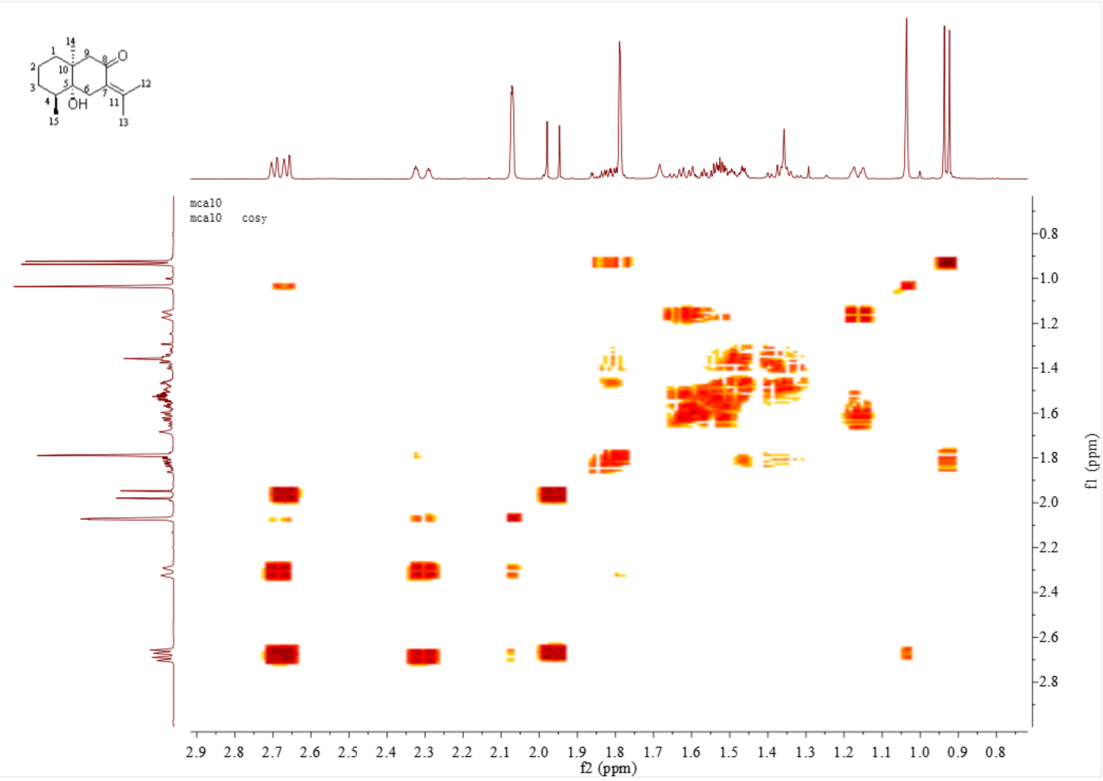

Figure S33. ^{1}H-^{1}H COSY (500 MHz, $CDCl_3$) spectrum of **5**.

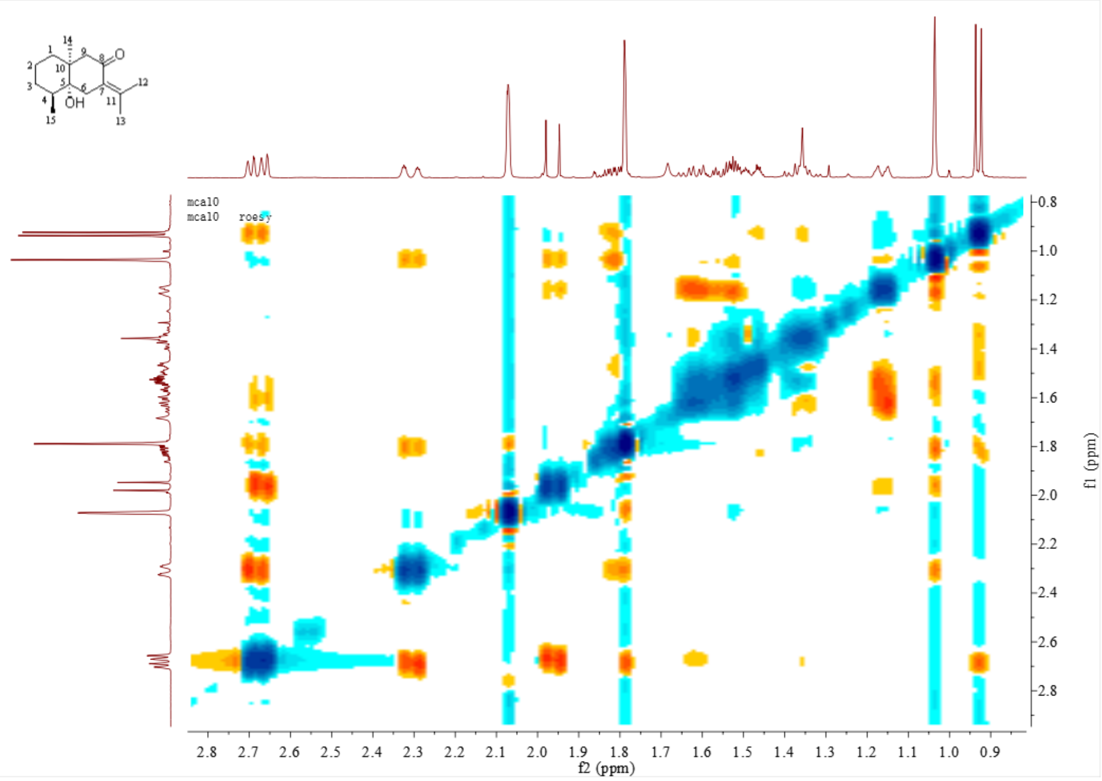

Figure S34. NOESY (500 MHz, $CDCl_3$) spectrum of **5**.

Formula Predictor Report - MCA-10.lcd Page 1 of 1

Data File: E:\DATA\2021\0720\MCA-10.lcd

Elmt	Val.	Min	Max	Elmt	Val.	Min	Max	Elmt	Val.	Min	Max	Elmt	Val.	Min	Max	Use Adduct
H	1	10	150	O	2	0	30	P	3	0	0	Se	2	0	0	Na
2H	1	0	0	F	1	0	0	S	2	0	0	Br	1	0	5	
B	3	0	0	Na	1	0	0	Cl	1	0	0	Pd	2	0	0	
C	4	10	150	Mg	2	0	0	Co	2	0	0	Ag	1	0	0	
N	3	0	10	Si	4	0	0	Cu	2	0	0	I	3	0	0	

Error Margin (ppm):	5	DBE Range:	not fixed	Electron Ions:	both
HC Ratio:	unlimited	Apply N Rule:	yes	Use MSn Info:	yes
Max Isotopes:	all	Isotope RI (%):	1.00	Isotope Res:	10000
MSn Iso RI (%):	75.00	MSn Logic Mode:	OR	Max Results:	20

Event#: 1 MS(E+) Ret. Time : 0.853 -> 0.893 Scan# : 129 -> 135

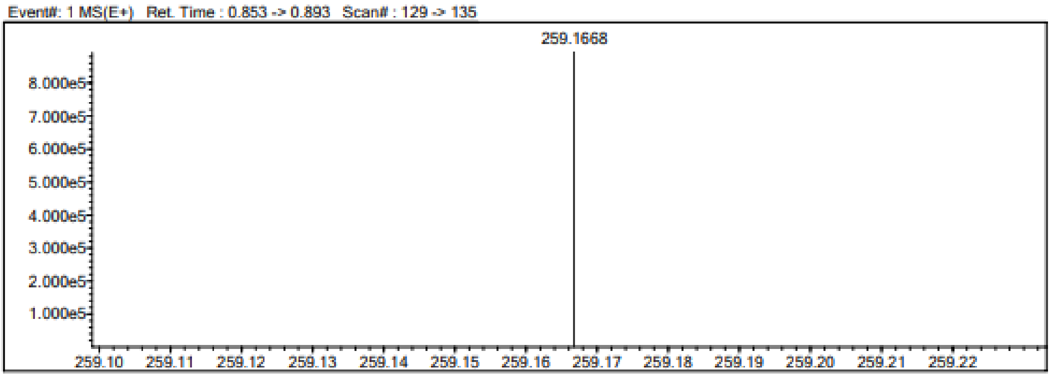

Measured region for 259.1668 m/z

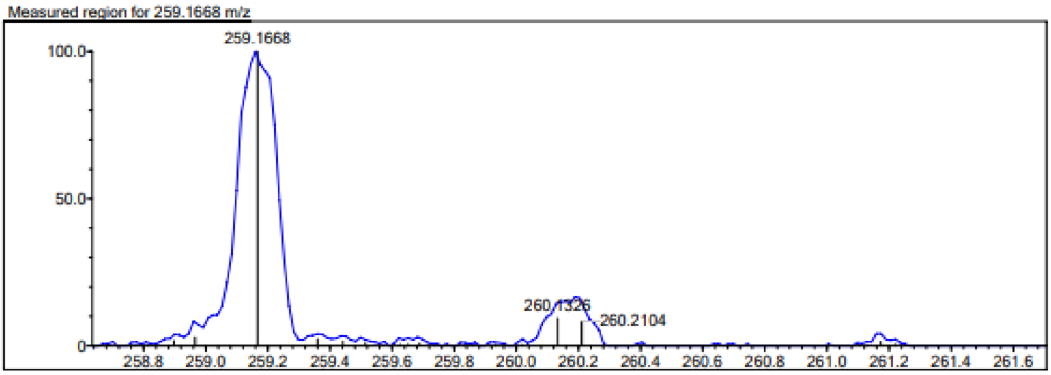

C15 H24 O2 [M+Na]+ : Predicted region for 259.1669 m/z

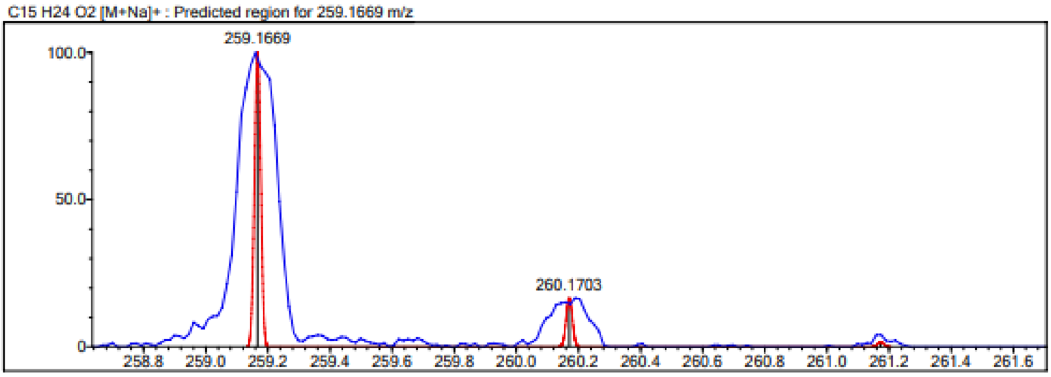

Formula (M)	Ion	Meas. m/z	Pred. m/z	Df. (mDa)	Df. (ppm)	DBE
C15 H24 O2	[M+Na]+	259.1668	259.1669	-0.1	-0.39	4.0

Figure S35. HR-ESI-MS spectrum of **5**.

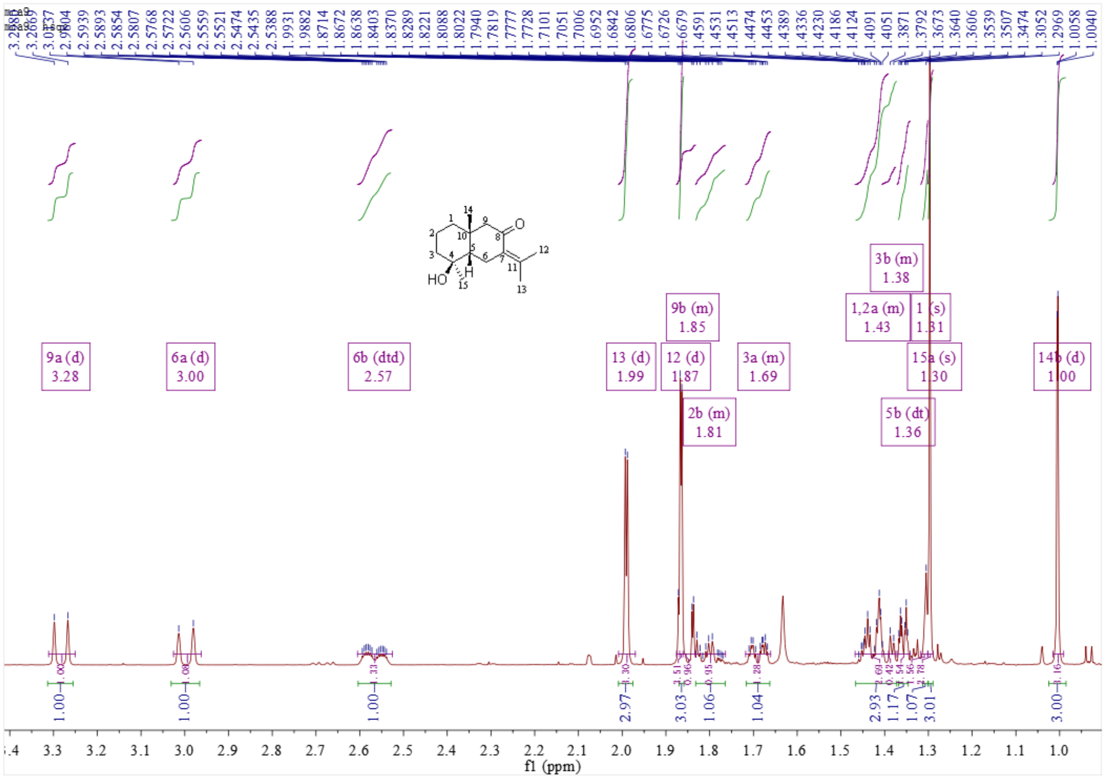

Figure S36. ^{1}H NMR (500 MHz, $CDCl_3$) spectrum of **6**.

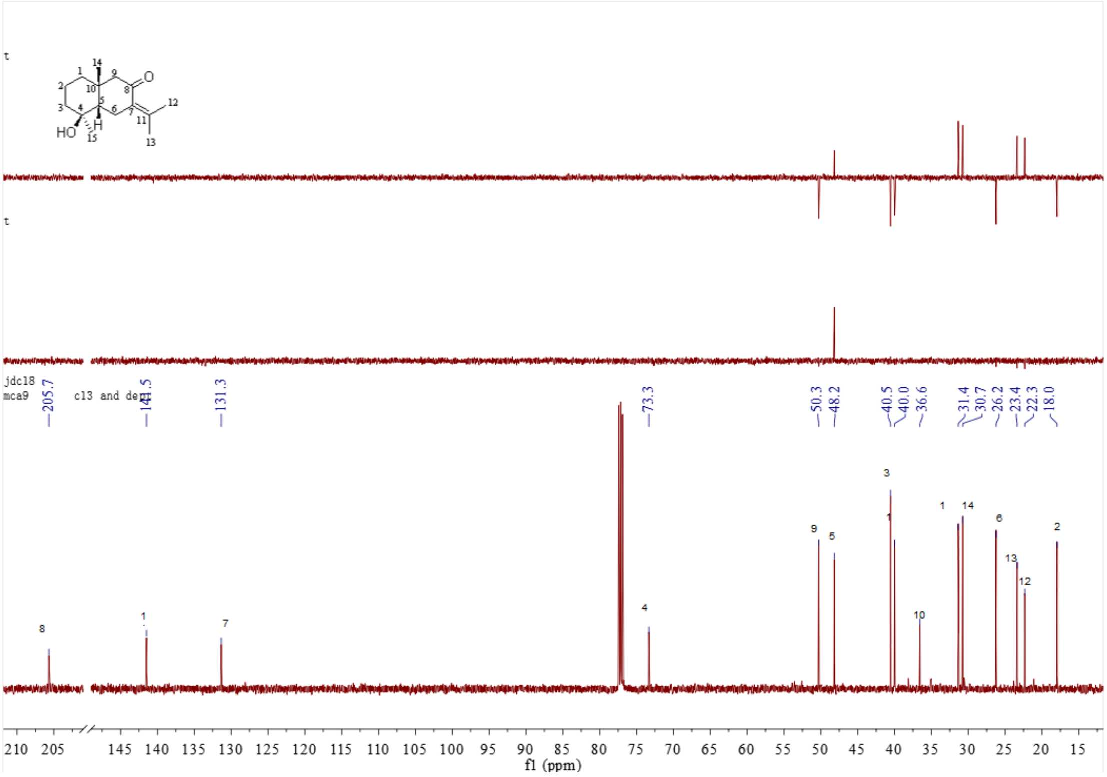

Figure S37. ^{13}C NMR (125 MHz, $CDCl_3$) spectrum of **6**.

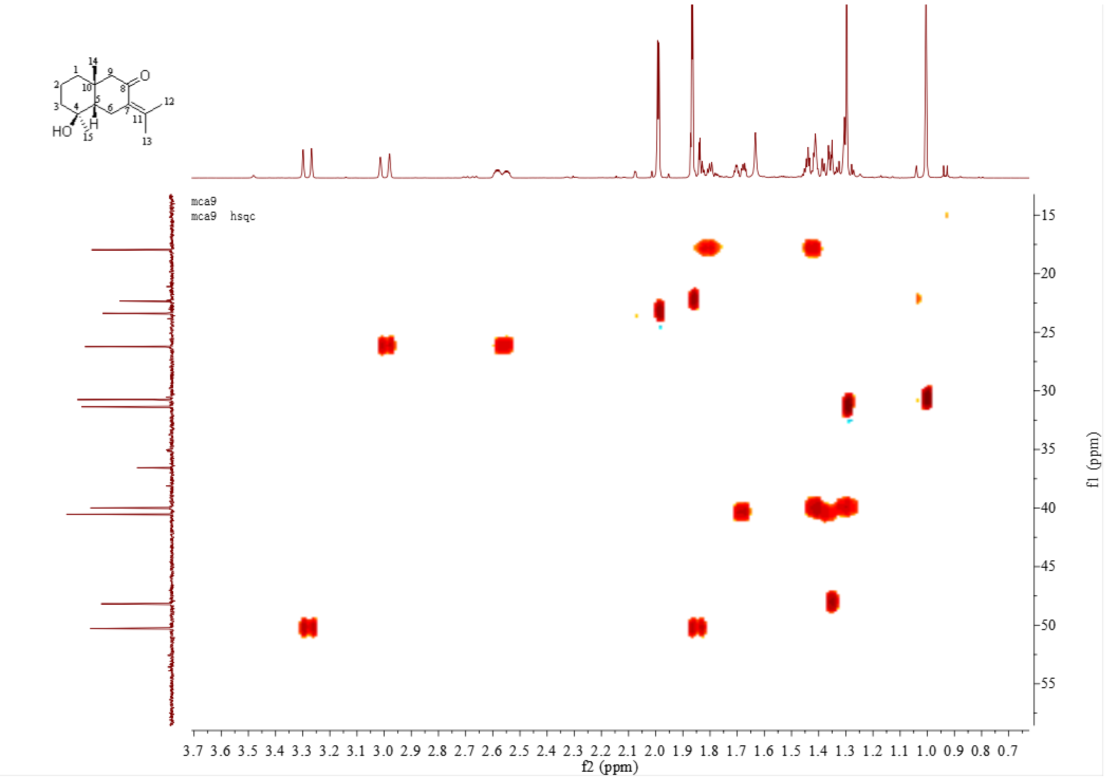

Figure S38. HSQC (500 MHz, CDCl3) spectrum of **6**.

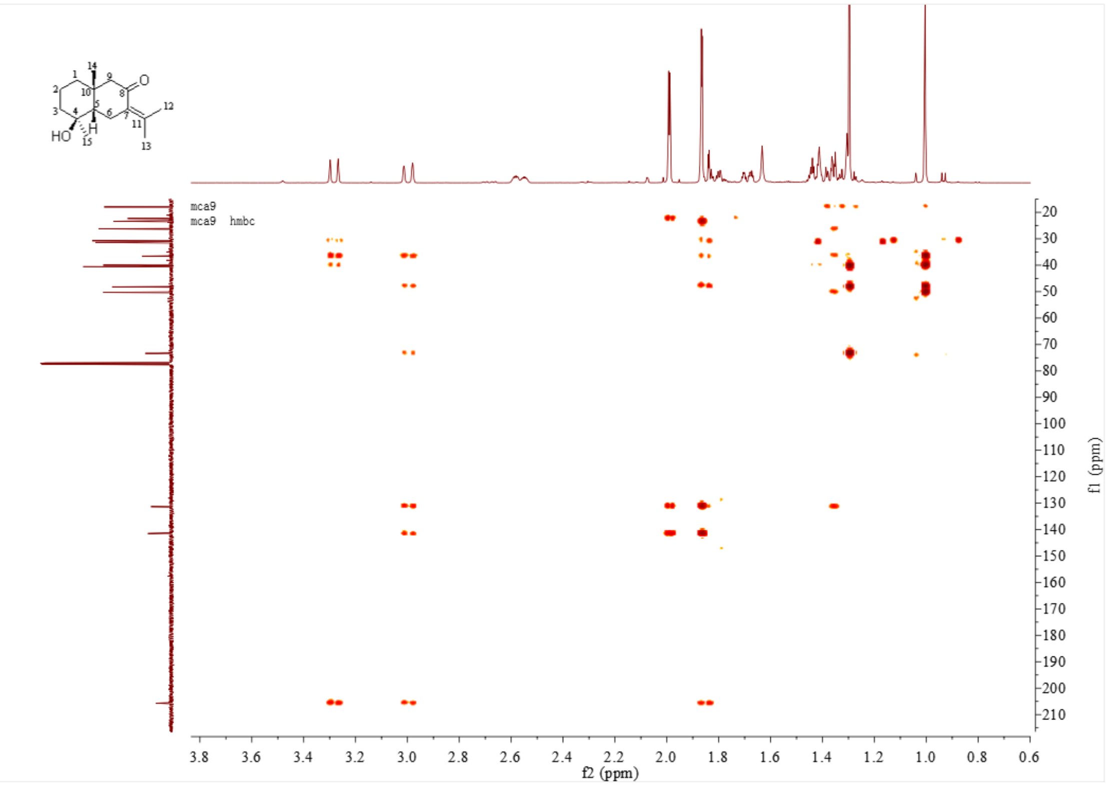

Figure S39. HMBC (500 MHz, CDCl3) spectrum of **6**.

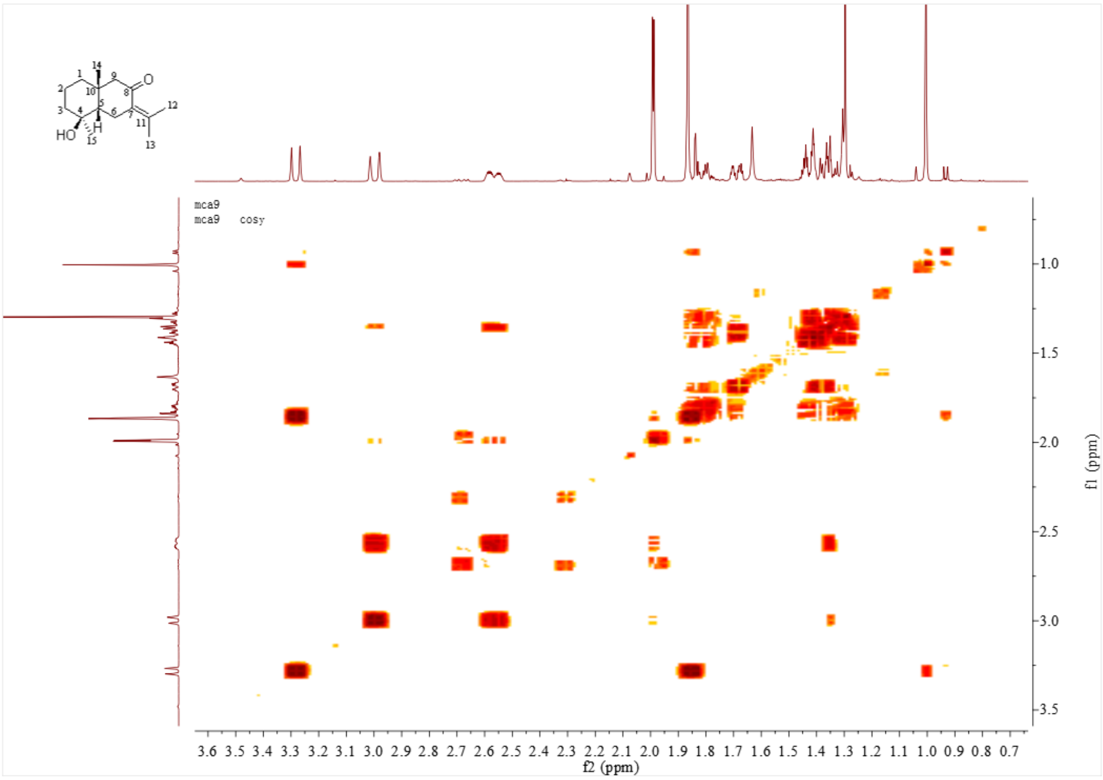

Figure S40. ^1H-^1H COSY (500 MHz, $CDCl_3$) spectrum of **6**.

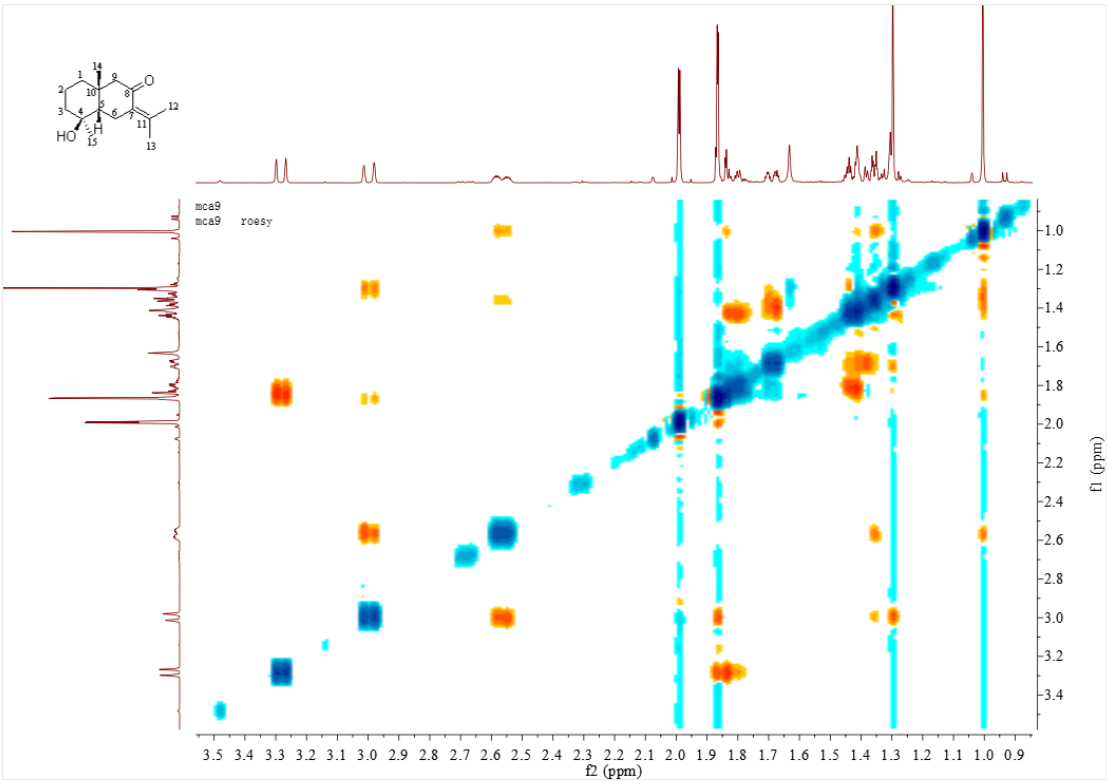

Figure S41. NOESY (500 MHz, $CDCl_3$) spectrum of **6**.

Formula Predictor Report - MCA-9.lcd Page 1 of 1

Data File: E:\DATA\2021\0720\MCA-9.lcd

Elmt	Val.	Min	Max	Elmt	Val.	Min	Max	Elmt	Val.	Min	Max	Elmt	Val.	Min	Max	Use Adduct
H	1	10	150	O	2	0	30	P	3	0	0	Se	2	0	0	Na
2H	1	0	0	F	1	0	0	S	2	0	0	Br	1	0	5	
B	3	0	0	Na	1	0	0	Cl	1	0	0	Pd	2	0	0	
C	4	10	150	Mg	2	0	0	Co	2	0	0	Ag	1	0	0	
N	3	0	10	Si	4	0	0	Cu	2	0	0	I	3	0	0	

Error Margin (ppm): 5
HC Ratio: unlimited
Max Isotopes: all
MSn Iso RI (%): 75.00

DBE Range: not fixed
Apply N Rule: yes
Isotope RI (%): 1.00
MSn Logic Mode: OR

Electron Ions: both
Use MSn Info: yes
Isotope Res: 10000
Max Results: 20

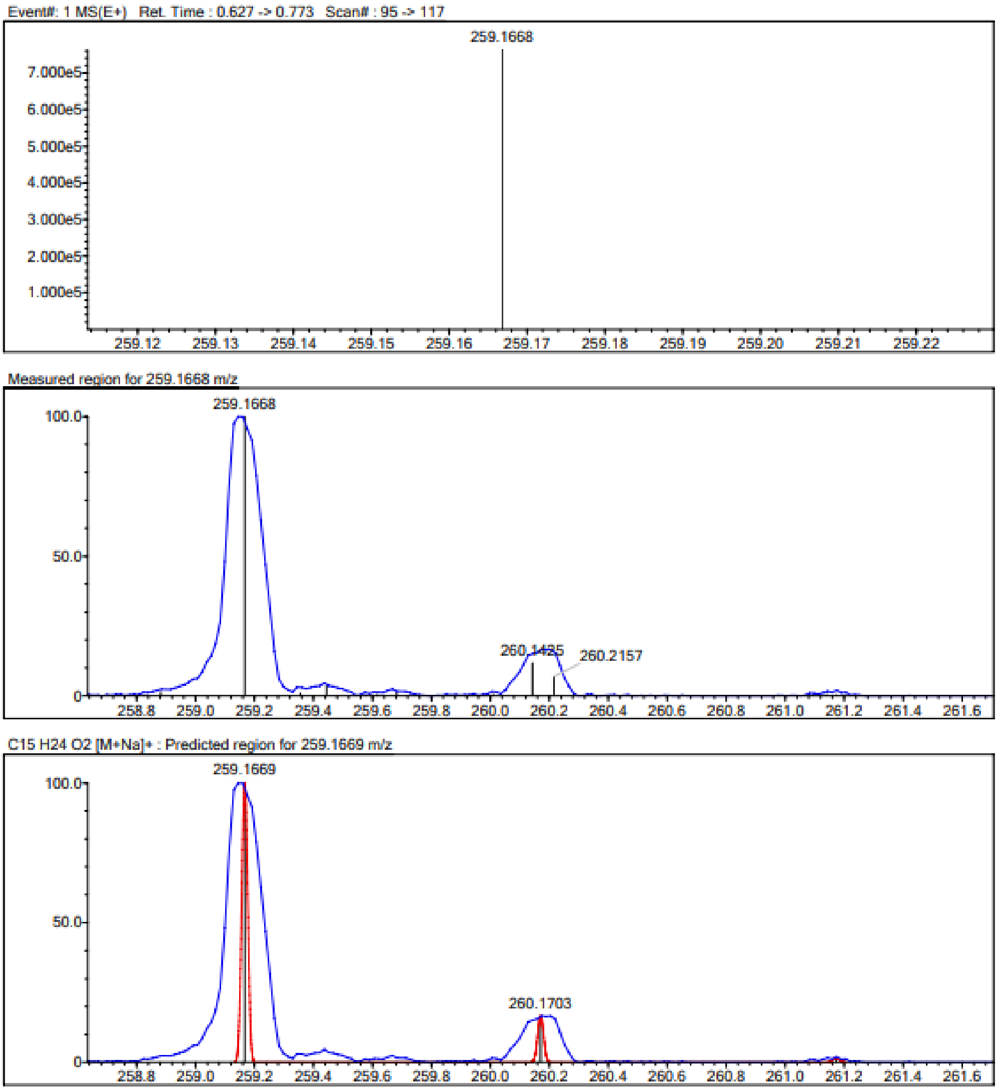

Formula (M)	Ion	Meas. m/z	Pred. m/z	Df. (mDa)	Df. (ppm)	DBE
C15 H24 O2	[M+Na]+	259.1668	259.1669	-0.1	-0.39	4.0

Figure S42. HR-ESI-MS spectrum of **6**.

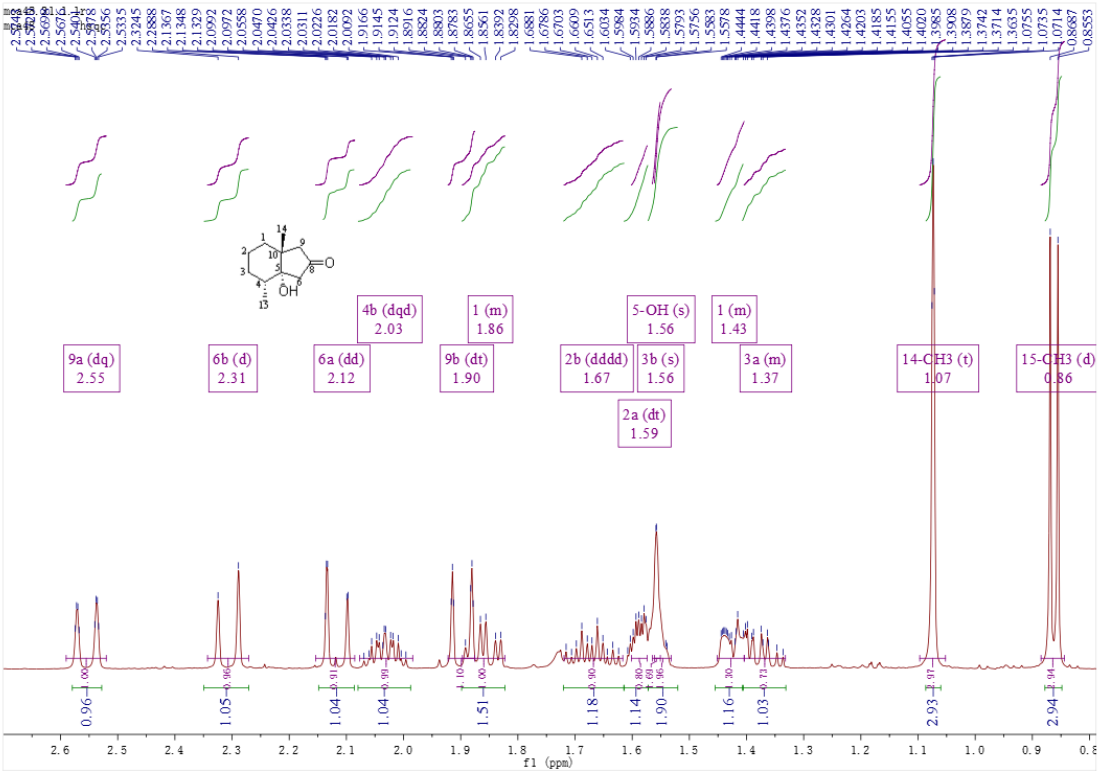

Figure S43. ^{1}H NMR (500 MHz, $CDCl_3$) spectrum of **7**.

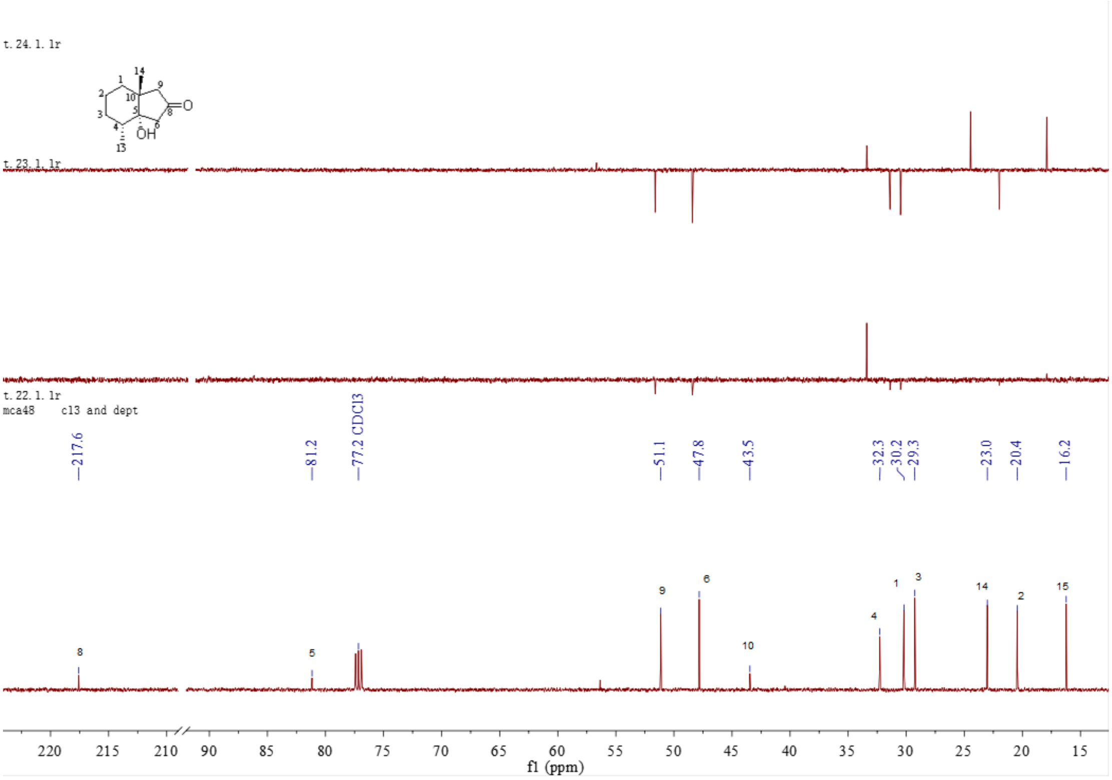

Figure S44. ^{13}C NMR (125 MHz, $CDCl_3$) spectrum of **7**.

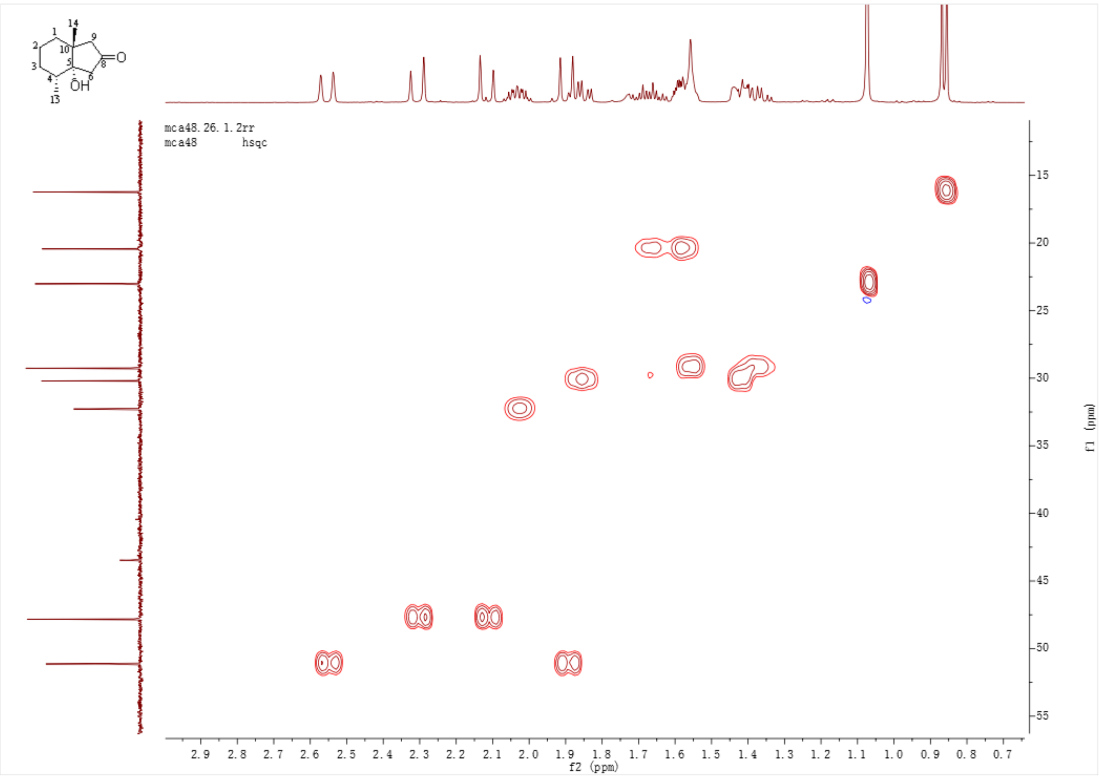

Figure S45. HSQC (500 MHz, CDCl3) spectrum of **7**.

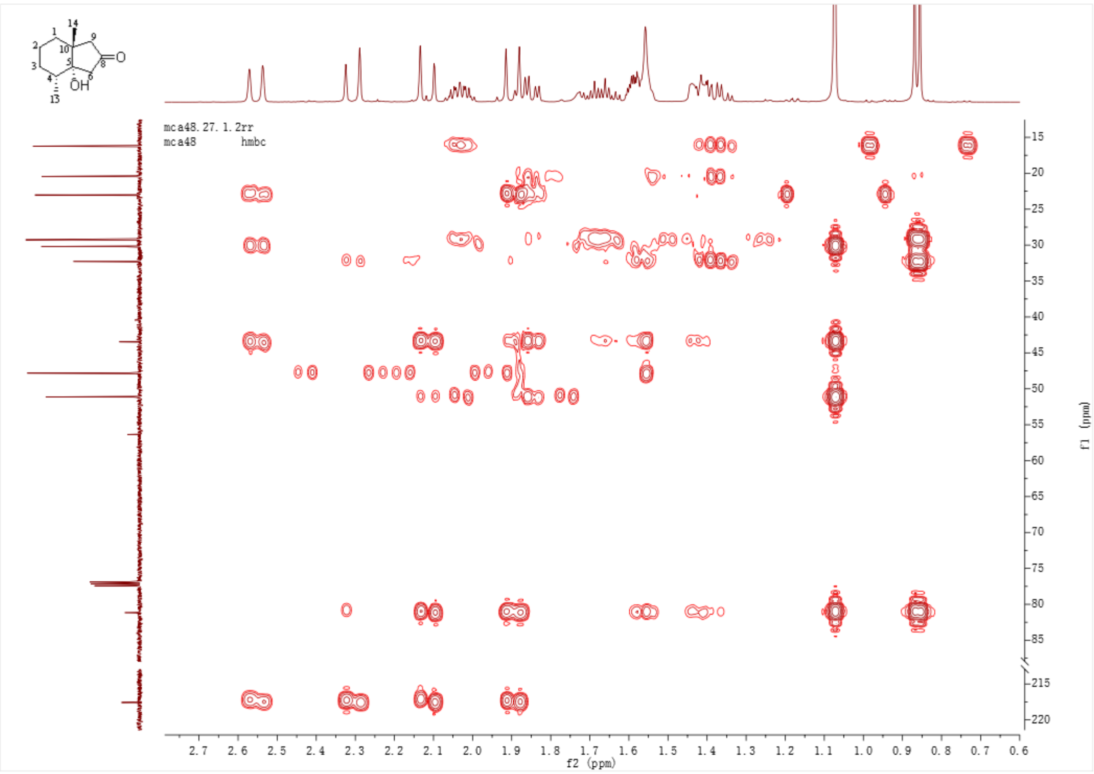

Figure S46. HMBC (500 MHz, CDCl3) spectrum of **7**.

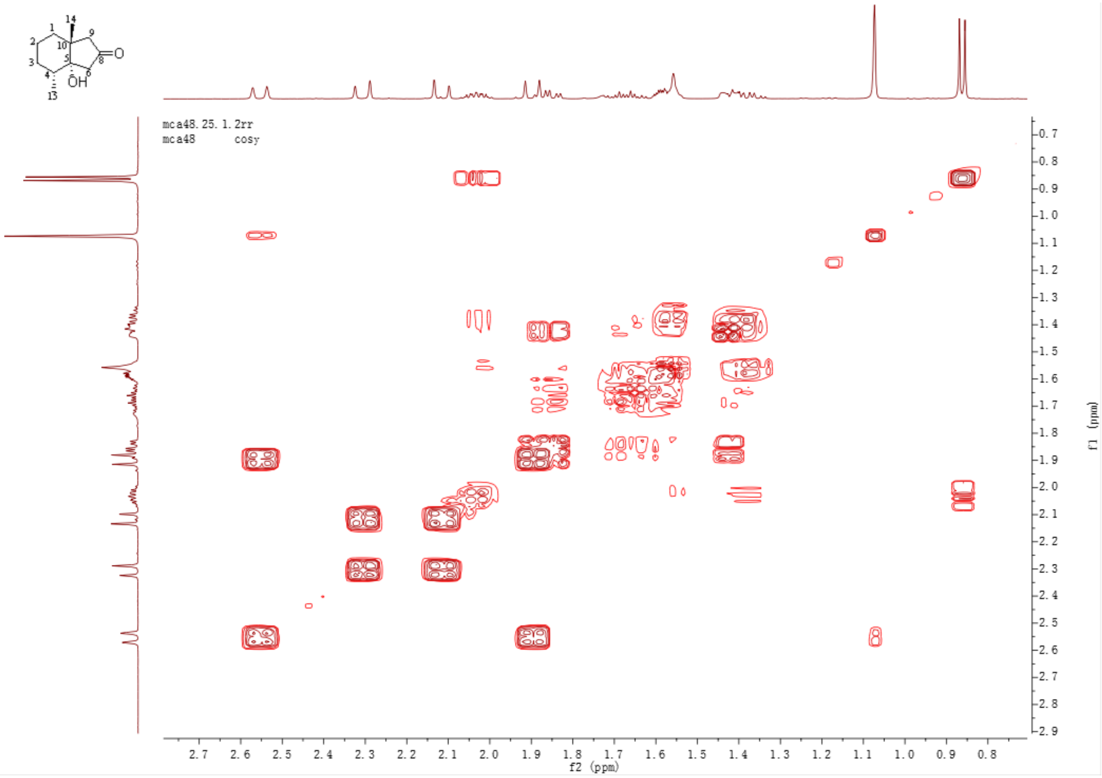

Figure S47. ^1H-^1H COSY (500 MHz, $CDCl_3$) spectrum of **7**.

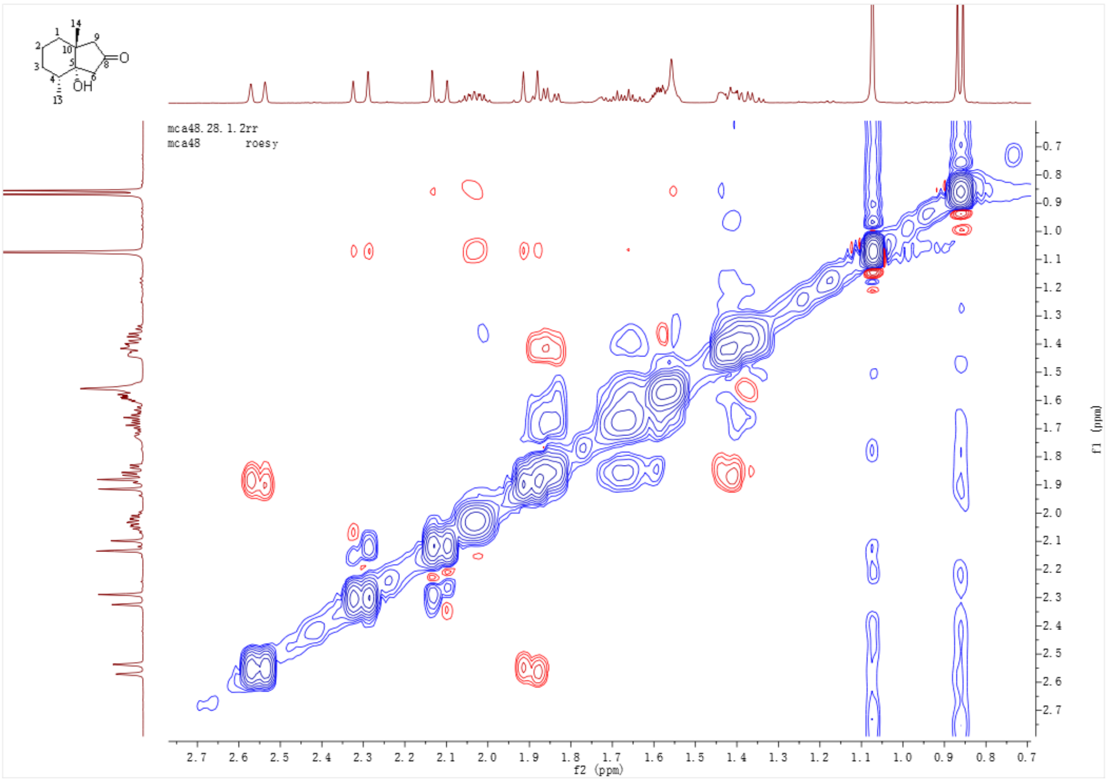

Figure S48. NOESY (500 MHz, $CDCl_3$) spectrum of **7**.

Formula Predictor Report - MCA-48.lcd Page 1 of 1

Data File: E:\DATA\2021\1206\MCA-48.lcd

Elmt	Val.	Min	Max	Elmt	Val.	Min	Max	Elmt	Val.	Min	Max	Elmt	Val.	Min	Max	Use Adduct
H	1	10	100	F	1	0	0	Cl	1	0	0	Ag	1	0	0	Na
2H	1	0	0	Na	1	0	0	Co	2	0	0	I	3	0	0	
B	3	0	0	Mg	2	0	0	Cu	2	0	0	Ir	3	0	0	
C	4	5	50	Si	4	0	0	Se	2	0	0					
N	3	0	10	P	3	0	0	Br	1	0	5					
O	2	0	30	S	2	0	0	Pd	2	0	0					

Error Margin (ppm): 5
HC Ratio: unlimited
Max Isotopes: all
MSn Iso RI (%): 75.00
DBE Range: not fixed
Apply N Rule: yes
Isotope RI (%): 1.00
MSn Logic Mode: OR
Electron Ions: both
Use MSn Info: yes
Isotope Res: 10000
Max Results: 20

Event#: 1 MS(E+) Ret. Time : 0.400 -> 0.427 Scan# : 61 -> 65

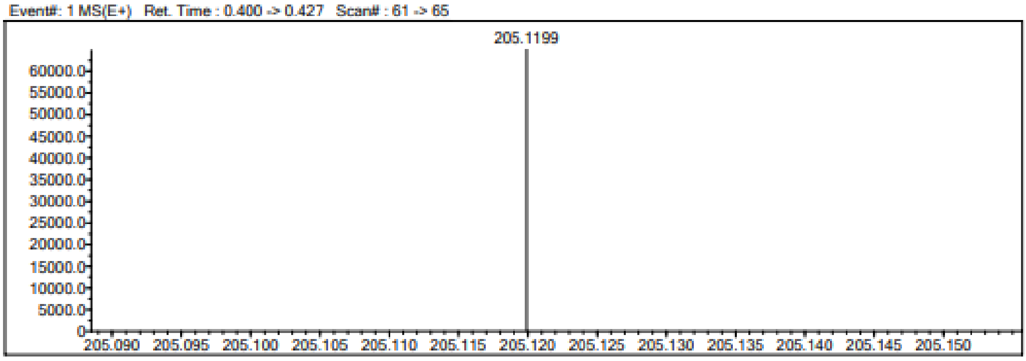

Measured region for 205.1199 m/z

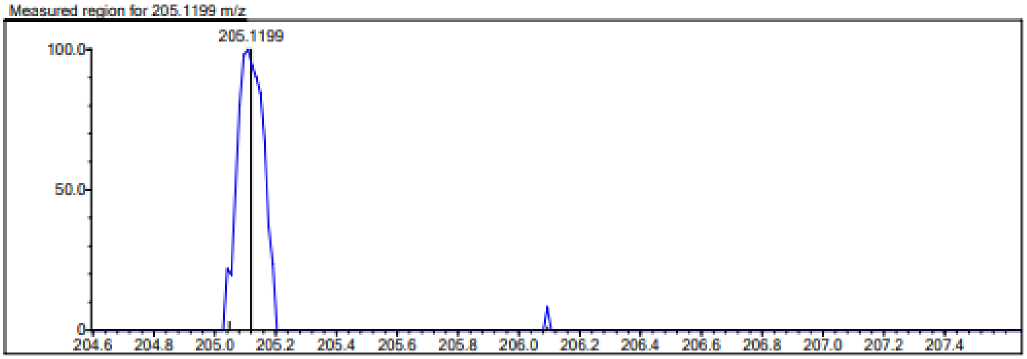

C11 H18 O2 [M+Na]+ : Predicted region for 205.1199 m/z

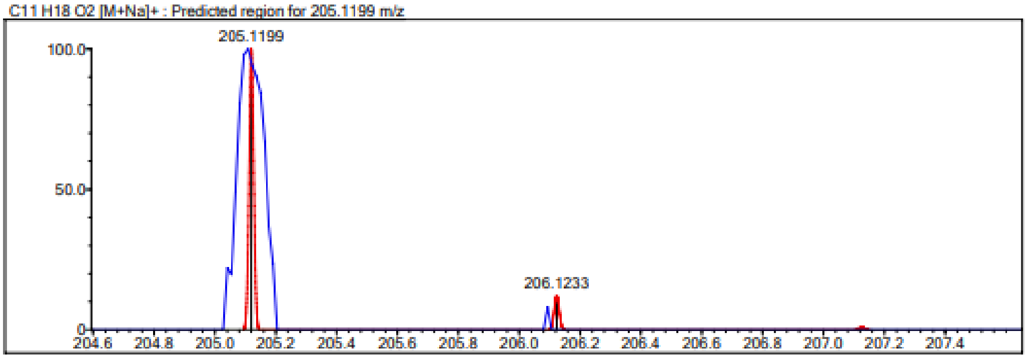

Formula (M)	Ion	Meas. m/z	Pred. m/z	Df. (mDa)	Df. (ppm)	DBE
C11 H18 O2	[M+Na]+	205.1199	205.1199	-0.0	0.00	3.0

Figure S49. HR-ESI-MS spectrum of **7**.
